# Genomic tagging of endogenous human ESCRT-I complex preserves ESCRT-mediated membrane-remodeling functions

**DOI:** 10.1074/jbc.RA119.009372

**Published:** 2019-09-13

**Authors:** Huxley K. Hoffman, Melissa V. Fernandez, Nicholas S. Groves, Eric O. Freed, Schuyler B. van Engelenburg

**Affiliations:** ‡ Molecular and Cellular Biophysics Program, Department of Biological Sciences, University of Denver, Denver, Colorado 80210; § Virus-Cell Interaction Section, HIV Dynamics and Replication Program, Center for Cancer Research, NCI-Frederick, National Institutes of Health, Frederick, Maryland 21702

**Keywords:** endosomal sorting complexes required for transport (ESCRT), human immunodeficiency virus (HIV), virus assembly, molecular cell biology, host-pathogen interaction, CRISPR/Cas, endogenous tag, membrane scission, method development, tumor susceptibility 101 (Tsg101)

## Abstract

The endosomal sorting complexes required for transport (ESCRT) machinery drives membrane scission for diverse cellular functions that require budding away from the cytosol, including cell division and transmembrane receptor trafficking and degradation. The ESCRT machinery is also hijacked by retroviruses, such as HIV-1, to release virions from infected cells. The crucial roles of the ESCRTs in cellular physiology and viral disease make it imperative to understand the membrane scission mechanism. Current methodological limitations, namely artifacts caused by overexpression of ESCRT subunits, obstruct our understanding of the spatiotemporal organization of the endogenous human ESCRT machinery. Here, we used CRISPR/Cas9-mediated knock-in to tag the critical ESCRT-I component tumor susceptibility 101 (Tsg101) with GFP at its native locus in two widely used human cell types, HeLa epithelial cells and Jurkat T cells. We validated this approach by assessing the function of these knock-in cell lines in cytokinesis, receptor degradation, and virus budding. Using this probe, we measured the incorporation of endogenous Tsg101 in released HIV-1 particles, supporting the notion that the ESCRT machinery initiates virus abscission by scaffolding early-acting ESCRT-I within the head of the budding virus. We anticipate that these validated cell lines will be a valuable tool for interrogating dynamics of the native human ESCRT machinery.

## Introduction

The endosomal sorting complexes required for transport (ESCRT)^2^ subunits make up an evolutionarily conserved membrane scission machinery responsible for diverse cellular processes that involve membrane budding away from the cytosol (1). In multivesicular body (MVB) formation, ESCRT proteins act at endosomes to sequester ubiquitinated receptors and other protein cargos and extrude the membrane into intraluminal vesicles to be delivered to the lysosome for degradation; this pathway for downregulating receptor-mediated signaling is important to cellular homeostasis and cell-to-cell communication (2–5). The ESCRTs also drive abscission between daughter cells in cytokinesis (6, 7), and many more roles of the ESCRT machinery have recently been identified, including autophagy (8, 9), immunological synapse formation (10, 11), extracellular vesicle release (12, 13), plasma membrane repair (14), neuron pruning (15), and lysosome repair (16). Dysfunctions of the ESCRT machinery lead to diseases such as cancers (17, 18) and neurodegeneration (9, 19, 20). Moreover, this cellular machinery is exploited by retroviruses such as HIV-1 and other enveloped viruses such as Ebola, which rely on the host ESCRT machinery to abscise virus particles from the surface of infected cells (21–25). Hijacking of the ESCRT machinery is critical to the replication of these viruses, and this host-pathogen interaction is not targeted by any of the current approved antiviral drugs, so it presents a potential target for new classes of antivirals (26–28). The many critical roles of the ESCRT machinery in human health and disease therefore make it imperative to understand the mechanisms of ESCRT function.

The ESCRT machinery is composed of several sequentially acting multiprotein complexes: the early-acting ESCRT-0, -I, and -II and the late-acting ESCRT-III and Vps4 (29, 30). For MVB biogenesis, the ESCRT machinery is engaged by ESCRT-0 binding to the endosomal lipid phosphatidylinositol 3-phosphate and recruiting ESCRT-I; for many of the other ESCRT functions, ESCRT-I is recruited by different upstream targeting factors, such as the centrosomal protein CEP55 for cytokinesis or the viral structural protein Gag for retrovirus budding. ESCRT-I recruits ESCRT-II, and all three early ESCRT complexes bind and sequester ubiquitinated cargos. ESCRT-II links the early and late ESCRT machinery by nucleating polymers of ESCRT-III on target membranes. The ESCRT accessory protein ALIX can also serve to recruit ESCRT-III. Upon targeting by these early ESCRT factors, membrane scission is performed by ESCRT-III filaments together with the AAA ATPase Vps4, which remodels and disassembles these filaments. Different ESCRT-dependent cellular functions use different subsets of the ESCRT machinery.

Although extensive work in yeast and *in vitro* has characterized the biochemical interactions between the ESCRT proteins, the spatial organization, dynamics, and mechanisms of the native ESCRT machinery in human cells still remain poorly characterized. For example, a major unresolved question about ESCRT-mediated abscission of membrane buds such as virus particles is the topology of ESCRT factors at abscission sites (31–34). Other open questions include the link between the early and late ESCRTs in processes such as HIV-1 release, for which the canonical bridging factor ESCRT-II appears to be dispensable (35–38), and the mechanism by which the late ESCRTs constrict and sever membrane necks (33, 34).

The lack of conclusive answers to fundamental questions about cellular ESCRT mechanisms is due in large part to limitations of the available imaging probes. Fluorescent protein (FP) tagging offers a means to address such questions by enabling measurement of the spatiotemporal organization of ESCRT proteins at target membranes inside living cells. A serious problem with this approach, however, is that exogenous expression of FP-tagged ESCRT subunits by transfection or transduction generally yields excess subunits (overexpression), which can lead to dominant negative artifacts and ESCRT dysfunction (39, 40). The presence of the untagged native ESCRT protein in the cells also makes it uncertain whether the behavior of an overexpressed FP-ESCRT probe accurately reflects the activity of the native protein (41). Indeed, dynamics of overexpressed FP-ESCRT probes have been shown in some cases to differ significantly from the dynamics of the endogenous ESCRTs (41). Using immunofluorescence to directly detect endogenous ESCRT subunits avoids these problems, but unfortunately, commercially available antibodies reliable enough for sensitive applications such as superresolution imaging are not available for most of the ESCRT proteins (33, 42, 43), and this approach also has the disadvantage of requiring fixation and permeabilization, which sacrifices dynamic information that can be obtained from living cells and can cause additional artifacts (44). Expression of an FP-tagged ESCRT protein from its native genomic locus would therefore be an optimal approach to allow imaging of the dynamics of the endogenous protein in living cells. However, because some FP fusions disrupt protein function even in the absence of overexpression (45), an endogenously FP-tagged ESCRT probe still requires rigorous validation to determine that the tag itself does not perturb ESCRT functions.

Whereas studies in genetically tractable model organisms have benefited tremendously from endogenous tagging, human tissue cultures have until recently been intractable for such approaches. The development of CRISPR-Cas9 as a programmable tool for site-specific gene editing (46, 47) has enabled FP-tagging of endogenous proteins in human cells (48). The Cas9 endonuclease generates a double-strand break (DSB) in the genomic DNA at a target site specified by a guide RNA. A homologous template can be integrated into the DSB by the process of homology-directed repair (HDR), allowing for “knock-in” of a transgene into the target site in the genome. Otherwise, the DSB is repaired by the more efficient process of nonhomologous end joining (NHEJ), leaving an insertion or deletion (indel), which can “knock out” expression of the targeted gene. Although use of the Cas9 gene editing technology for gene knockout in human cells has become widespread, generation of viable human cell lines with knock-in tags is more challenging, requiring careful design and optimization of both the guide RNA and the HDR template.

Here, we applied CRISPR-Cas9 and HDR-mediated knock-in (KI) to endogenously tag the core ESCRT-I subunit Tsg101, which recruits the downstream ESCRT machinery to its targeting factors for most of the known ESCRT functions. We present cell lines expressing Tsg101 with a GFP tag at physiological or lower levels from its native genomic locus in two widely used human cell types, HeLa epithelial cells and Jurkat T cells. We show that the GFP-Tsg101 KI does not disrupt the function of ESCRT machinery in cytokinesis, MVB biogenesis, or HIV-1 budding, indicating that the GFP tag is nonperturbing. We then demonstrate the utility of this probe to address open questions about ESCRT mechanisms by using it to measure the incorporation of endogenous Tsg101 in released HIV-1 particles, supporting a model in which the ESCRT machinery initiates virus abscission by scaffolding early-acting ESCRT-I within the head of the budding virus. These validated GFP-Tsg101 KI cell lines offer a valuable new tool for researchers to interrogate the spatiotemporal organization of the endogenous ESCRT machinery in human cells.

## Results

### Generation of GFP-Tsg101 knock-in cell lines

**Figure 1.**
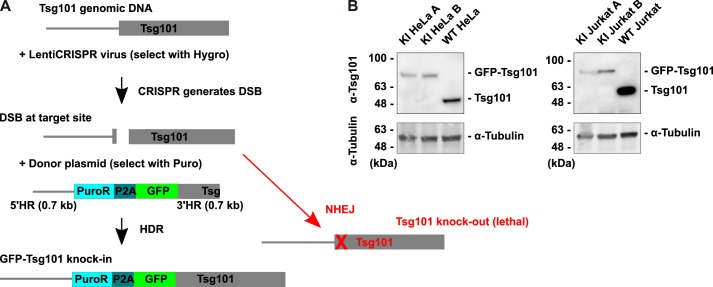
**GFP-Tsg101 knock-in using CRISPR-Cas9 and HDR.** *A*, knock-in strategy. Cells were transduced with LentiCRISPRv2 to express Cas9 with sgRNA targeting the start of the *Tsg101* gene and transfected with a donor template plasmid containing a puromycin resistance selection marker and a GFP tag flanked by HRs for the target site. Cas9 generates a DSB in the genomic DNA at the target site, and the donor template can be integrated into the DSB by HDR, resulting in GFP knock-in. Otherwise, the DSB is repaired by the more efficient process of NHEJ, leaving insertions or deletions that knock out Tsg101. Tsg101 knockout was lethal, which helped to select against cells that did not achieve the knock-in. *B*, Western blotting for Tsg101 in cell lysates of GFP-Tsg101 KI clonal lines and WT parental lines. The KI cell lines express GFP-Tsg101 at lower than WT levels and do not express untagged Tsg101. α-Tubulin is shown as a loading control.

We used CRISPR-Cas9 and HDR to introduce a GFP tag at the N terminus of endogenous Tsg101 (Fig. 1*A*) in two immortalized human cell lines: HeLa epithelial cells, which are widely used throughout cell biology, and Jurkat T cells, which are widely used in immunology and in the study of HIV replication. The CRISPR components, Cas9 endonuclease and single guide RNA (sgRNA) targeting a site near the 5′-end of the *Tsg101* gene (Fig. S1*A*), were expressed in cells by lentiviral transduction, using LentiCRISPRv2 (49) modified to carry a hygromycin-resistance selection marker. To generate the HeLa and Jurkat GFP-Tsg101 KI, cells were transfected or electroporated, respectively, with HDR template donor plasmid prior to lentiviral delivery of the CRISPR components. The HDR template (Fig. S1*B*) coded for a puromycin-resistance selection marker and a GFP tag linked by a P2A self-cleaving peptide, flanked by ∼700-bp regions of homology for the insertion site at the start of the *Tsg101* gene, with silent mutations to disrupt the Tsg101 sgRNA target sequence in the 3′ homology region. Because the 5′ homology region contains a portion of the endogenous *Tsg101* promoter sequence (50), we found that a small fraction of the cell population possessed off-target integrations of the HDR template that yielded puromycin resistance and GFP fluorescence; however, cells that did not integrate the HDR template on-target to repair their *Tsg101* allele were usually knocked out for Tsg101, which led to lethality and selection for on-target KI. Similar results were observed in both HeLa and Jurkat cell lines, demonstrating that these observations were not cell type–dependent. Clonal lines of GFP-Tsg101 KI cells were isolated from the populations that survived selection. To help control for clonal variation or any off-target CRISPR effects, many of the subsequent validation experiments were performed on two clonal KI lines (designated KI A and KI B) of each cell type.

We analyzed Tsg101 protein expression in these clonal KI cell lines by Western blotting for Tsg101 in cell lysates (Fig. 1*B*). All clonal KI lines tested expressed GFP-Tsg101 and no untagged Tsg101; the absence of untagged Tsg101 is important to ensure that the tagged form reports on all activity of Tsg101 in the cell and allows for quantification of protein expression levels based on GFP fluorescence. The levels of GFP-Tsg101 in the KI cell lines were lower than the levels of Tsg101 in the parental cell lines, confirming that the KI lines avoided the problems of overexpression. Sequencing of the genomic target site revealed that the KI cell lines were heterozygous for GFP-Tsg101, with one allele having the GFP insertion and the other allele having a knockout indel (Fig. S2). This indicated that a single functional *Tsg101* allele was sufficient for cell survival, and because NHEJ occurs much more efficiently than HDR (51), the second allele was then much more likely to be a knockout. Sequencing of the KI alleles showed that the integration at the homology junctions was precise (Fig. S3). Furthermore, Western blotting for GFP in cell lysates demonstrated that the only GFP in the KI cell lines was the full-length GFP-Tsg101 fusion protein (Fig. S4), indicating both that the GFP-Tsg101 fusion is stable and that the KI cells do not express any additional GFP from off-target integrations of the HDR template.

### Functional validation of GFP-Tsg101 knock-in cell lines

**Figure 2.**
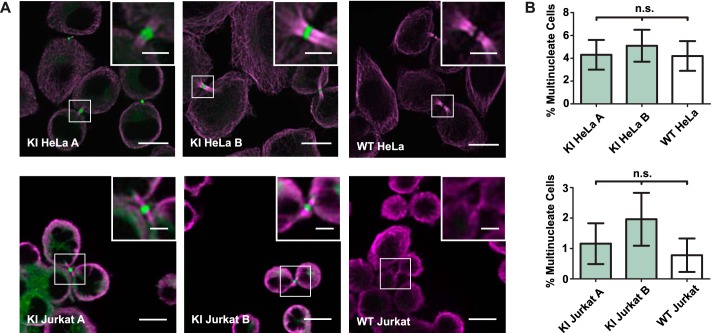
**Endogenous GFP-Tsg101 localizes to midbodies during cytokinesis, and cytokinesis is not disrupted in knock-in cell lines.** *A*, to test the participation of GFP-Tsg101 in cytokinetic abscission, dividing cells were immunostained for α-tubulin (*magenta*) to highlight cytokinetic bridges and imaged by confocal fluorescence microscopy. GFP-Tsg101 (*green*) localized to midbody structures, indicating that the GFP tag did not block the recruitment of Tsg101 for cytokinesis. *Scale bars*, 10 μm; *inset scale bars*, 3 μm. *B*, to assess whether the knock-in disrupted cytokinetic abscission, cell lines were scored for multinuclearity (*n* > 250 cells/sample) as a measure of the rate of failure of cytokinesis. There was no significant difference between the multinucleate rates for the GFP-Tsg101 KI clones and WT parental control of each cell type, as assessed by one-way analysis of variance (*p* = 0.8724 for HeLa, *p* = 0.4869 for Jurkat), indicating that the expression of GFP-Tsg101 in the KI cell lines was sufficient for cytokinesis. *n.s.*, not significant; *Error bars*, S.E.

We proceeded to assess the function of the endogenously expressed GFP-Tsg101 in the ESCRT-dependent processes of cytokinetic abscission, MVB biogenesis, and HIV-1 release. First, to address whether endogenously expressed GFP-Tsg101 participated in cell division and daughter cell abscission, we observed cells undergoing the final stages of cytokinesis. We found that endogenous GFP-Tsg101 localized to midbodies during cytokinesis (Fig. 2*A*), as observed previously for exogenously expressed Tsg101 (6, 7). Furthermore, depletion of Tsg101 has been shown to cause significant cytokinesis defects resulting in an increased proportion of multinucleate cells (6, 7). To address whether the tagging of endogenous Tsg101 impaired cytokinetic abscission, we scored the KI cell lines for the frequency of multinucleate cells (Fig. 2*B*). GFP-Tsg101 KI HeLa and Jurkat lines showed no significant increase in multinuclearity compared with the parental lines, indicating that the expression of GFP-Tsg101 in these cells did not disrupt ESCRT-mediated cytokinetic abscission.

**Figure 3.**
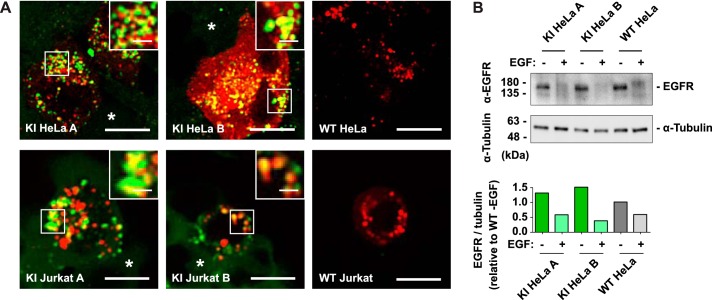
**Endogenous GFP-Tsg101 localizes to MVBs and does not impair ESCRT-mediated EGFR degradation.** *A*, to test the recruitment of GFP-Tsg101 to endosomes, cells were transfected with both mCherry-Vps4-DN (*red*) and untagged Vps4-DN to stall ESCRT disassembly. Confocal fluorescence microscopy shows GFP-Tsg101 (*green*) concentrated on the swollen stalled endosomes (*insets*) in transfected cells, in contrast to its mainly cytosolic distribution in untransfected cells (*). This indicates that the GFP tag does not block the interaction of Tsg101 with the other components of the ESCRT pathway in MVB biogenesis. *Scale bars*, 10 μm; *inset scale bars*, 2 μm. *B*, to assess ligand-induced degradation of EGFR, serum-starved cells were stimulated with 100 ng/ml EGF for 3 h (+) or left unstimulated (−), and EGFR levels were assessed by Western blotting of cell lysates (*top*) and quantitatively compared by densitometry of the Western blotting (*bottom*), with a blot for α-tubulin used to normalize for loading. EGF stimulation resulted in a robust decrease in EGFR for the GFP-Tsg101 KI HeLa clones as well as for WT HeLa control, showing that the ESCRT-dependent process of EGFR degradation is preserved in the KI cell lines.

Next, we tested the function of the endogenously tagged Tsg101 in the ESCRT-mediated MVB pathway. Tsg101 and other ESCRT components are mainly cytosolic at steady state, transiently assembling on endosomal membranes to perform MVB cargo sorting and intraluminal vesicle formation and then being disassembled and released back to the cytosol by the action of the ATPase Vps4. To verify that GFP tagging of endogenous Tsg101 does not prevent its interface with the ESCRT machinery at sites of MVB biogenesis, we transfected cells with a dominant-negative mutant of Vps4 (Vps4-DN), which blocks the disassembly step, leading to accumulation of ESCRTs on endosomes (52). In both HeLa and Jurkat cell lines, endogenous GFP-Tsg101 localized to arrested MVB/endosomal structures induced by expression of Vps4-DN (Fig. 3*A*), confirming that the GFP tag did not sterically occlude its interaction with the other ESCRT components in MVB biogenesis. To evaluate whether the tagging of endogenous Tsg101 impaired the function of the ESCRT machinery in endosomal trafficking, we then tested the trafficking of a well-characterized ESCRT-dependent MVB cargo, the epidermal growth factor receptor (EGFR). The action of the ESCRT machinery at MVBs is key to the ligand-induced degradation of receptors such as EGFR, with depletion of Tsg101 leading to inhibition of EGFR degradation (53). We therefore tested the degradation of EGFR following stimulation by its ligand EGF. This assay was performed only in the HeLa cell lines because Jurkat T cells do not express EGFR (54). EGF stimulation led to loss of EGFR in the GFP-Tsg101 KI HeLa cells as in the parental cells (Fig. 3*B*), showing that the tagging of endogenous Tsg101 did not impair its function in endosomal trafficking for receptor down-regulation.

**Figure 4.**
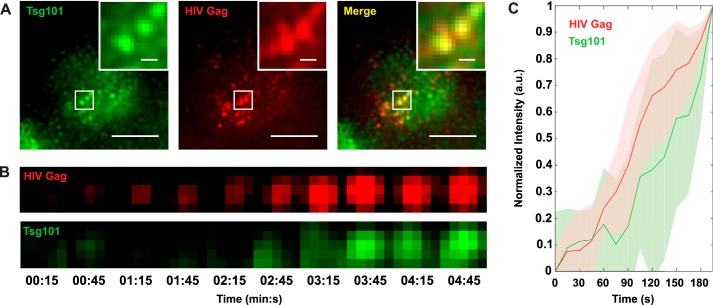
**Endogenous GFP-Tsg101 is co-recruited with HIV-1 Gag to sites of virus assembly.** To test the recruitment of GFP-Tsg101 by HIV-1 Gag, GFP-Tsg101 KI HeLa cells (clone A) were infected with HIV-1 expressing a TagRFP-tagged nanobody probe that labels Gag, and the live cells were imaged by TIRF-M. *A*, GFP-Tsg101 colocalizes with Gag at virus assembly sites on the plasma membrane of an infected cell, indicating that the GFP tag did not block the Tsg101-Gag interaction. *Scale bars*, 10 μm; *inset scale bars*, 1 μm. *B*, Tsg101 and Gag intensity growth over time at an individual virus assembly site. Pixel size was 108.33 nm. *C*, mean normalized fluorescence intensity in arbitrary units (*a.u.*) for Tsg101 and Gag over time at individual virus assembly sites (*n* = 5). Tsg101 accumulation at these sites is coincident with Gag accumulation, indicating that Tsg101 is progressively co-recruited along with Gag during virus assembly. *Shaded regions*, S.D.

We next assessed the function of the endogenously tagged Tsg101 in HIV-1 replication. HIV-1 budding from infected cells relies on direct recruitment of Tsg101 by the viral structural protein Gag to engage the host cell ESCRT machinery and abscise budding virus particles from the plasma membrane (21, 23). To demonstrate the recruitment of GFP-Tsg101 for virus budding, cells were infected with HIV-1 (NL4-3 strain) expressing a nanobody reporter that labels HIV-1 Gag (55) fused to the red fluorescent protein TagRFP (56). Live-cell total internal reflection fluorescence microscopy (TIRF-M) showed that GFP-Tsg101 colocalizes with HIV-1 Gag on the plasma membrane of infected cells (Fig. 4*A*). Taking advantage of the high spatial and temporal resolution of this approach, we analyzed the fluorescence intensity over time at individual virus assembly sites, finding that Tsg101 progressively co-accumulated at these sites along with Gag (Fig. 4, *B* and *C*). These results confirmed that the GFP tag on Tsg101 does not interfere with the Tsg101-Gag interaction.

**Figure 5.**
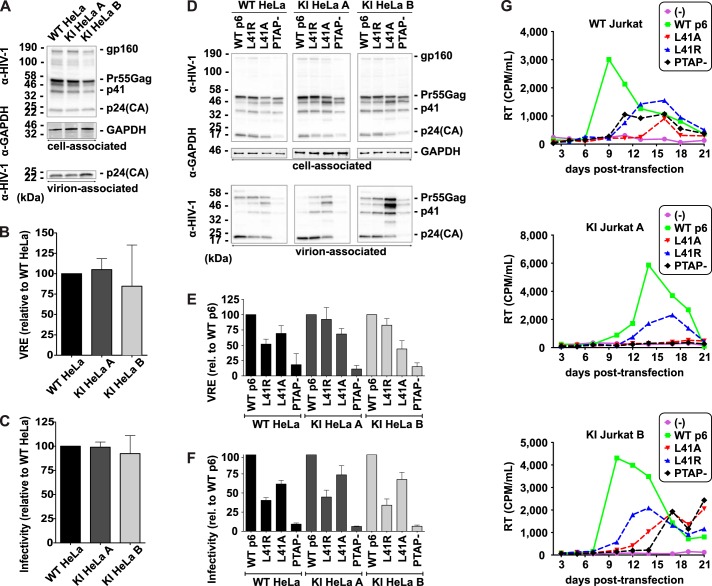
**HIV-1 release and replication are not perturbed in GFP-Tsg101 knock-in cell lines.** *A–C*, GFP-Tsg101 knock-in does not alter HIV-1 particle release and infectivity. For assessment of HIV-1 release and infectivity, HeLa cells were transiently transfected with the HIV-1 molecular clone pNL4-3. *A*, virus release assay. Cell and virion lysates were analyzed by SDS-PAGE and Western blotting for HIV-1 proteins, followed by chemiluminescent analysis. Positions of the HIV-1 Env glycoprotein precursor gp160, the HIV-1 Gag precursor Pr55Gag, the Gag processing intermediate p41, and HIV-1 p24(CA) are shown. GAPDH is shown as a loading control. *B*, virus release efficiencies (*VRE*) were calculated as the amount of virion-associated p24(CA) as a fraction of the total (cell plus virion) Gag protein detected and normalized to the result for WT HeLa; *n* = 3; mean ± S.D. (*error bars*). *C*, infectivity assay. Virus produced by HeLa clones was normalized for RT activity and used to infect TZM-bl cells, and luciferase activity was measured 2 days postinfection. *n* = 3; mean ± S.D. *D–F*, GFP-Tsg101 knock-in does not alter the Tsg101 dependence of HIV-1 particle release and infectivity. To assess the contributions of Tsg101 and ALIX, HeLa cells were transiently transfected with the WT HIV-1 molecular clone pNL4-3 (WT p6) or the L41A, L41R, or PTAP− p6 mutants. *D*, virus release assay, performed as in *A. E*, virus release efficiencies were calculated as in *B* and normalized to the result for WT p6 for each cell line; *n* = 3; mean ± S.D. *F*, infectivity assay, performed as in *C*; *n* = 3; mean ± S.D. For the KI HeLa lines as for WT HeLa, PTAP− severely reduced the infectivity of the released virus, whereas L41A and L41R gave lesser defects in infectivity, showing that the dependence of HIV-1 infectivity on Tsg101 was not altered by the knock-in. *G*, GFP-Tsg101 knock-in does not disrupt HIV-1 replication in T cells. Spreading infection assays were performed in parental or knock-in Jurkat T cells transfected with WT or p6-mutant pNL4-3 molecular clones. Cultures were split, and supernatants were collected for RT analysis every 2–3 days.

To test whether GFP-Tsg101 KI impaired HIV-1 replication, we measured the efficiency of HIV-1 particle release and infectivity. We determined that the GFP-Tsg101 KI cell lines were unimpaired for virus release (Fig. 5 (*A* and *B*) and Fig. S5 (*A* and *B*)) and that virus particles produced from these cells were as infectious as those produced from control cells (Fig. 5*C*). While Tsg101 is the primary route by which HIV-1 Gag hijacks the ESCRT machinery, HIV-1 also has a secondary route for recruitment of the downstream ESCRTs, via the ESCRT-associated protein ALIX (24, 57, 58). ALIX normally plays a lesser role than Tsg101 in HIV-1 release, but overexpression of ALIX can rescue the virus release defect caused by loss of the Tsg101-Gag interaction (58–60). Tsg101 and ALIX are recruited by distinct motifs in the p6 domain of HIV-1 Gag, so to rule out the possibility that HIV-1 budding in the GFP-Tsg101 KI lines could be relying on ALIX instead of Tsg101, we tested the release and infectivity of HIV-1 with mutations in these motifs: the PTAP− mutation (61) removes the Tsg101-binding site (21, 62), whereas the L41A and L41R mutations disrupt the ALIX-binding site (24, 57, 63, 64). For the GFP-Tsg101 KI HeLa lines as for WT HeLa, the p6-PTAP− mutation severely reduced virus release (Fig. 5 (*D* and *E*) and Fig. S5 (*A* and *C*)) and infectivity (Fig. 5*F*), whereas the p6-L41A and -L41R mutations yielded much milder defects in release and infectivity (Fig. 5, *D–F*), consistent with published results (64). These results indicate that the differential roles of Tsg101 and ALIX in HIV-1 release were not perturbed by the KI. In Jurkat T cells, spreading infections (Fig. 5*G*) showed that the KI did not prevent HIV-1 from spreading in culture. The p6 mutants were also delayed in their ability to spread relative to WT p6 in the KI Jurkat lines as in the parental Jurkat line, further demonstrating that the KI did not alter the relative contributions of Tsg101 and ALIX in HIV-1 replication. There was some variation in the peak of infection between the cell lines; this variation, however, appears to be unrelated to Tsg101, because the KI Jurkat lines were not impaired for particle release (Fig. S5, *A* and *B*), which is the Tsg101-dependent step of HIV-1 replication. Because the KI cell lines are single-cell clones, some variation in the kinetics of infection is expected due to clonal variability in other host cell factors involved at other stages of HIV-1 replication.

To further rule out the possibility of ALIX substituting for Tsg101 functions in the KI cells, we also examined ALIX expression levels (Fig. S6). Up-regulation of ALIX expression in the KI lines would suggest that ESCRT-I function was impaired and that cells had compensated by relying more heavily on ALIX to recruit ESCRT-III. Western blotting showed that this was not the case, as the KI lines expressed ALIX at similar levels as the parental lines.

### Imaging endogenous Tsg101 in released HIV-1 particles

**Figure 6.**
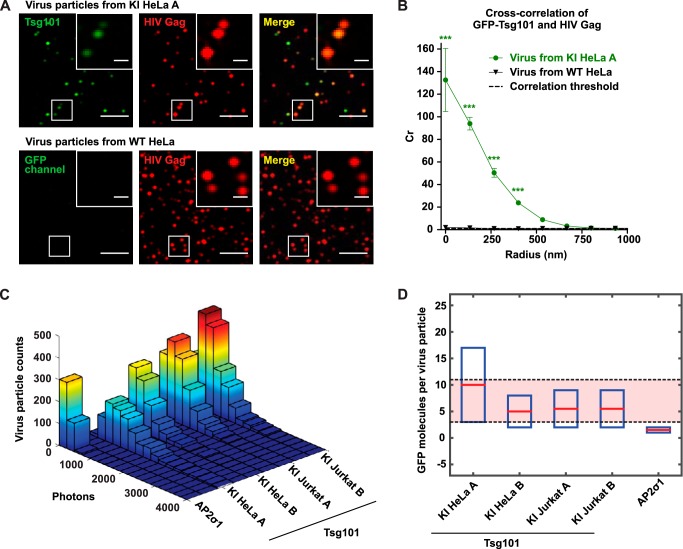
**Endogenous GFP-Tsg101 is robustly and specifically incorporated into HIV-1 particles, with ∼3–11 GFP-Tsg101 molecules incorporated per virion.** *A*, for imaging of released HIV-1 particles, GFP-Tsg101 KI HeLa A cells, and WT HeLa control were infected with HIV-1 expressing a TagRFP-tagged nanobody that labels HIV-1 Gag (*red*). Virus particles released from these cells were harvested 2 days after infection, purified by sucrose cushion ultracentrifugation, digested with subtilisin to remove all proteins not protected within the Gag lattice of a virus particle, and imaged by TIRF-M. The virus sample from the GFP-Tsg101 KI cells showed GFP-Tsg101 (*green*) within released HIV-1 particles (*insets*). *Scale bars*, 5 μm; *inset scale bars*, 1 μm. *B*, cross-correlation (*C_r_*) analysis of released virus particle images demonstrates strong positive spatial correlation of Tsg101 and HIV-1 Gag. *C_r_* values greater than 1 reflect positive correlation. *Error bars*, S.E.; *n* = 875 particles for KI HeLa A; *n* = 997 particles for WT HeLa. *C_r_* for virus particles from GFP-Tsg101 KI cells showed a statistically significant difference (***, *p* < 0.0001) from *C_r_* for virus particles from WT cells. *C*, HIV-1 particles were prepared and imaged as above from each of the GFP-Tsg101 KI HeLa and Jurkat clonal lines and from HeLa cells stably expressing GFP-AP2σ1 as a control to test for nonspecific incorporation. Histograms of GFP photon counts per virus particle show that all four GFP-Tsg101 KI virus samples contained higher levels of GFP than the GFP-AP2σ1 control virus sample, indicating specific HIV-1 incorporation of Tsg101. Photon counts per HIV-1 particle (range from mean of lognormal distribution to mean + 3 S.D.) were 1184–3992 for KI HeLa A, 661–1949 for KI HeLa B, 851–2068 for KI Jurkat A, 753–1967 for KI Jurkat B, and 217–546 for GFP-AP2σ1 HeLa. *D*, numbers of GFP molecules per HIV-1 particle were calculated by comparing GFP photon counts with a single-molecule GFP standard (see Fig. S7). Virus particles from each cell line were independently assessed, and lower and upper bounds for the numbers of GFP molecules per virus particle were calculated from the mean and mean + 3 S.D. photon counts, respectively. The approximate number of GFP-Tsg101 molecules in HIV-1 particles from GFP-Tsg101 KI cell lines ranged from 3 to 11 (*red-shaded region*), whereas HIV-1 particles from GFP-AP2σ1 HeLa were estimated to contain only 1–2 molecules of GFP-AP2σ1 passively incorporated.

Having validated the functionality of the GFP-Tsg101 KI cell lines, we applied them to tackle a major uncertainty about the organization of ESCRT-I at sites of HIV-1 assembly. An unresolved question is whether the ESCRT machinery drives virus abscission from within the head of the virus bud, in which case some ESCRT components would be trapped in the virus particle after release from the cell, or from the cytosolic side of the bud neck, in which case the ESCRTs would remain in the cell after virus release (33, 34). To address this question, we tested whether the endogenously expressed GFP-Tsg101 was present in released HIV-1 particles. HeLa cells were infected with HIV-1 (NL4-3) expressing a TagRFP-tagged nanobody that labels HIV-1 Gag. TIRF-M imaging of purified released virus from GFP-Tsg101 KI HeLa cells showed GFP-Tsg101 in the HIV-1 particles (Fig. 6*A*). Cross-correlation analysis of the images confirmed the extremely strong colocalization of GFP-Tsg101 with HIV-1 Gag in virus particles (Fig. 6*B*). This experimental approach is important, as any contamination from shed microvesicles (13) that contain luminal ESCRT components could be interpreted as associating with virus particles directly in bulk biochemical experiments. Direct imaging of purified virus particles conclusively demonstrates compartmentalization of GFP-Tsg101 within HIV-1 Gag particles. We then used this approach to quantify the number of endogenous GFP-Tsg101 molecules that are required to mediate virus abscission and further compared HIV-1 particle incorporation of Tsg101 across cell types. We analyzed the GFP photon counts per HIV-1 particle in images of purified released virus from each of the GFP-Tsg101 KI HeLa and Jurkat clonal lines (Fig. 6*C*). The difference in GFP intensity per HIV-1 particle between HeLa and Jurkat cell types was less than the difference between the two HeLa clonal lines, suggesting that HIV-1 particle incorporation of Tsg101 was not cell type–dependent. As a control to test for nonspecific passive incorporation, we also analyzed the GFP intensity in purified released HIV-1 particles from HeLa cells stably expressing a GFP-tagged adaptor protein complex 2 σ subunit (AP2σ1), a protein that acts at the plasma membrane but is not thought to be involved in HIV-1 particle abscission. All four GFP-Tsg101 KI virus samples showed higher levels of GFP per virus particle than the GFP-AP2σ1 control, indicating specific HIV-1 incorporation of Tsg101 relative to the passive incorporation that might be expected due to presence of Tsg101 at the plasma membrane. Finally, we compared the GFP photon counts from virus particles to a single-molecule GFP standard (Fig. S7) to quantitatively estimate the number of GFP-Tsg101 molecules incorporated (Fig. 6*D*). The approximate number of GFP-Tsg101 molecules in HIV-1 particles from GFP-Tsg101 KI cell lines ranged from 3 to 11, whereas we estimated HIV-1 particles from GFP-AP2σ1 HeLa to only contain 1–2 molecules of GFP-AP2σ1 passively incorporated.

## Discussion

The ESCRT membrane-remodeling machinery is key to many biomedically important cellular processes, including cell division, receptor down-regulation, and viral replication. The spatial organization, dynamics, and activities of the ESCRT proteins in human cells remain unclear due to limitations of available imaging probes. CRISPR-Cas9 and HDR-mediated KI has recently enabled fluorescent tagging of endogenously expressed human proteins. Herein, we describe GFP tagging of the endogenous core ESCRT-I component Tsg101 in human epithelial cell and T-cell lines. We rigorously validated these GFP-Tsg101 KI cell lines by demonstrating that the functions of the ESCRT machinery in cytokinetic abscission (Fig. 2), MVB biogenesis (Fig. 3), and HIV-1 replication (Figs. 4 and 5) were not significantly disrupted when compared with their respective parental lines. These GFP-Tsg101 KI cell lines enable live-cell imaging of native rather than overexpressed ESCRT machinery, representing a major improvement over previously available ESCRT probes.

We gave special attention to characterizing the interaction of Tsg101 with HIV-1 Gag in our system, because the essential role of Tsg101-Gag binding in HIV-1 replication makes it a potential target for new classes of antiretroviral therapy (27, 28). Similarly, because a number of other enveloped viruses (*e.g.* Ebola virus) rely on ESCRT machinery for particle budding, Tsg101 could be targeted in the context of other antiviral therapies. We measured the progressive co-accumulation of the endogenously tagged Tsg101 with Gag at individual virus assembly sites in live cells (Fig. 4), showing that GFP-Tsg101 arrives at the same time as Gag during assembly, rather than after Gag assembly is complete; this result corroborates previous findings that relied on exogenous expression of GFP-Tsg101 (32). We also conclusively demonstrated not only that HIV-1 release was unimpaired by the KI, but also that the dependence of viral release on Tsg101 in KI cells was unchanged (Fig. 5 and Fig. S5). Furthermore, we show that cell lines did not compensate for the introduction of a GFP tag into the endogenous Tsg101 protein by up-regulating ALIX (Fig. S6), which can in some contexts substitute for Tsg101 in recruiting downstream ESCRT components to membranes (7, 58). Although much research in virology and cell biology has been performed in HeLa cells, cell type differences make it important to study HIV-1 replication in the immune cell types that it natively infects. We therefore also developed the GFP-Tsg101 KI probe in Jurkat T cells. Our KI Jurkat cell lines thus offer a particularly valuable tool for HIV-1 research.

As an example of the utility of the GFP-Tsg101 KI, we applied this tool to interrogate the fate of ESCRT-I after ESCRT-mediated HIV-1 release from infected cells. The direct interaction between Tsg101 and Gag makes it plausible that Tsg101 would localize in the head of a virus bud and remain trapped in the virus particle after its release from the cell, which some previous studies have supported (31, 64); however, other studies have suggested that Tsg101 localizes to the neck of the virus bud (32, 66). In this study, the GFP-Tsg101 KI enabled us for the first time to image endogenous Tsg101 within cell-free virions (Fig. 6). Whereas HIV-1 particle incorporation of endogenous ESCRT-I has previously been demonstrated by Western blotting of the purified virus fraction (65), the direct imaging of endogenous Tsg101, made possible by gene editing, allows us to conclusively show that the GFP-Tsg101 containing particles in this fraction are HIV-1 Gag particles as opposed to extracellular vesicles. The presence of endogenous Tsg101 in released virus particles supports a model of ESCRT-I scaffolding directly to the Gag lattice, within the head of the budding HIV-1 particle, to mediate HIV-1 release. The KI furthermore allowed us to quantify the number of molecules of endogenous Tsg101 incorporated in each HIV-1 particle (Fig. 6*D*). Our results indicated ∼3–11 molecules of endogenous GFP-Tsg101 per virion, suggesting that a small number of ESCRT-I complexes are needed to nucleate downstream abscission activity. It is important to note that these estimates for copy number could be susceptible to environmental factors that influence the quantity of fluorophores estimated, but the limited number of ESCRT-I complexes per virion suggests that a fundamental stoichiometry of ESCRT-I is templated with the assembling Gag lattice. Further studies will be required to determine how this stoichiometry is maintained, considering that thousands of ESCRT-I–interacting motifs are presented by the Gag lattice at a single assembly site.

Although we have focused here on the three most studied ESCRT functions of cytokinesis, MVB biogenesis, and HIV-1 budding, many more roles of the ESCRTs have been identified, adding to the need for validated probes to equip researchers to explore new ESCRT functions. Different ESCRT functions use different subsets of the ESCRT proteins, but Tsg101 is the hub for ESCRT recruitment for the majority of ESCRT-dependent processes identified to date, making the GFP-Tsg101 KI cell lines particularly useful tools. ESCRT-mediated cellular functions shown to involve Tsg101 include release of exosomes (67) and microvesicles (13), repair of endolysosomes (16), neuron pruning (15), autophagy (8, 9), and formation of immunological synapses (10, 11). Besides HIV-1, other retroviruses such as human T-cell leukemia virus (68), filoviruses such as Ebola (22), and some arenaviruses, rhabdoviruses, and reoviruses (25) similarly hijack the ESCRTs via Tsg101 to enable virus release. Tsg101 also acts as a transcriptional regulator, both directly and by interaction with nuclear hormone receptors, and some of these transcriptional regulation functions appear to be independent of the ESCRT membrane scission machinery (69, 70). Collectively, our GFP-Tsg101 KI probe offers an improved means to interrogate these diverse functions.

In short, we have generated and validated GFP-Tsg101 KI human cell lines as a tool enabling fluorescent imaging of endogenously expressed ESCRT-I at its sites of action in live human cells. Because the ESCRT machinery is crucial in a multitude of cellular processes and in the replication of many viruses, this tool can facilitate advances in numerous biomedically important areas of research in cell biology and virology.

## Experimental procedures

### Cell culture

HeLa (CCL-2) and Jurkat (clone E6-1, TIB-152) cell lines and the HEK293T (CRL-11268) cell line used for lentiviral vector production were obtained from ATCC (Manassas, VA). TZM-bl cells (71) used for HIV-1 infectivity assays were obtained from J. Kappes though the National Institutes of Health AIDS Research and Reference Reagent Program (Germantown, MD). Cells were cultured in complete growth medium at 37 °C with 5% CO_2_. Complete growth medium was prepared with 10% fetal bovine serum (catalog no. 35-011-CV), 2 mm l-glutamine (catalog no. 25-005-CI), and 1× penicillin-streptomycin (catalog no. 30-002-CI) in Dulbecco's modified Eagle's medium (catalog no. 17-205-CV) for HeLa, HEK293T, and TZM-bl cells or in RPMI (catalog no. 17-105-CV) for Jurkat cells; the cell culture reagents were obtained from Corning, Inc. (Corning, NY).

### Plasmids and cloning

For the HDR template donor plasmid, the 5′ and 3′ homology regions (HRs) were amplified by PCR from human genomic DNA, these segments were joined to an EGFP segment by Gibson assembly (72), and this 5′HR-EGFP-3′HR cassette was cloned into a pN1 vector backbone (Clontech/Takara Bio USA, Mountain View, CA) using the NotI and AatII sites. A unique NdeI restriction site was added between the 5′HR and EGFP by site-directed mutagenesis, and the coding sequence for puromycin resistance followed by a P2A self-cleaving peptide was cloned into this site by Gibson assembly. Silent mutations were made to the sgRNA target site in the 3′HR to prevent CRISPR from cutting the HDR template.

The LentiCRISPRv2 transfer plasmid was a gift from Feng Zhang (Addgene plasmid 52961). The sgRNA was synthesized as oligonucleotides, phosphorylated and annealed, and ligated into the BsmBI site of LentiCRISPRv2. A hygromycin resistance gene was then inserted in place of the puromycin resistance gene by Gibson assembly into the BamHI site.

For expression of dominant negative Vps4 (Vps4-DN), Vps4A(E228Q), and Vps4B(E235Q), plasmids were created by cloning the human Vps4A or Vps4B cDNA into the 5′ XhoI and 3′ NotI sites of the pEGFP-N1 vector (Clontech), thus completely removing the EGFP sequence, and then introducing the E228Q or E235Q mutation, respectively, by site-directed mutagenesis. The mCherry-Vps4A(E228Q) plasmid was created by cloning the human Vps4A cDNA into a pmCherry-C1 vector backbone (Clontech) in frame with mCherry using 5′ HindIII and 3′ KpnI sites and then introducing the E228Q mutation by site-directed mutagenesis.

The transfer plasmid for expression of HIV-1 (NL4-3) with a TagRFP-tagged nanobody that labels HIV-1 Gag, referred to below as “pSV-dE-CANTD-TagRFP-3′LTR,” was cloned as follows. First, for single-round infectious HIV-1, the NL4-3 reference genome was cloned into an SV40 ori-containing backbone (pN1 vector; Clontech) for use as a transfer plasmid, and the *Env* gene was deleted by removal of the NsiI (6530)-BglII (7611) fragment spanning the majority of the gp120 coding region (NL4-3 numbering as in Ref. 73). The pCANTD-EGFP plasmid, coding for a camelid single-domain antibody (nanobody) that binds the CA domain of HIV-1 Gag (55) in fusion with EGFP, was a kind gift from Heinrich Leonhardt. TagRFP was cloned into the pCANTD-EGFP plasmid in place of EGFP, and then this CANTD-TagRFP cassette was cloned into the NL4-3 genome at the start of the Nef ORF by Gibson assembly into the XhoI site. The full-length HIV-1 molecular clone pNL4-3 (74) and the PTAP−, L41R (61), and L41A (63) mutants have been described previously.

For expression of GFP-AP2σ1, cDNA of human AP2 σ subunit isoform 5 was synthesized as a gBlocks Gene Fragment (Integrated DNA Technologies, Coralville, IA) and cloned by Gibson assembly into the AgeI site at the N terminus of GFP in the pLJM1-EGFP lentiviral transfer plasmid (75), which was a gift from David Sabatini (Addgene plasmid 19319).

### Lentivirus production

Replication-incompetent lentiviruses were produced by transfecting HEK293T cells with the lentiviral transfer plasmid along with the psPAX2 packaging plasmid (gift from Didier Trono, Addgene plasmid 12260) and pVSV-G pseudotyping plasmid (gift from Dr. Xuedong Liu, University of Colorado, Boulder, CO), using PEI transfection reagent (Alfa Aesar/Thermo Fisher Scientific, Tewksbury, MA, catalog no. 43896). The virus was harvested around 48 h after transfection, 0.45-μm-filtered, snap-frozen in liquid nitrogen, and stored at −80 °C.

### Generation of GFP-Tsg101 KI HeLa cell lines

HeLa cells at 60% confluence in a well of a 6-well plate were transfected with 2 μg of HDR template donor plasmid, using PEI transfection reagent. 4 h after transfection, cells were transduced with 1 ml of lentivirus of LentiCRISPRv2-Tsg101sgRNA-Hygro. Cells were selected with 500 μg/ml hygromycin starting 1 day after transfection and transduction and with 2 μg/ml puromycin starting 9 days after transfection and transduction. Following selection, clonal populations were isolated by plating cells at a series of dilutions to obtain well-separated colonies. After 9 days of growth, single colonies were trypsinized and transferred to wells of a 24-well plate to be cultured and expanded.

### Generation of GFP-Tsg101 KI Jurkat cell lines

2 × 10^5^ Jurkat cells were electroporated with 1.5 μg of HDR template donor plasmid, using the Neon Transfection System (Invitrogen) with 10-μl tip, 1325-V pulse voltage, 10-ms pulse width, and 3 pulses. 4 h after electroporation, cells were transduced with 0.5 ml of lentivirus of LentiCRISPRv2-Tsg101sgRNA-Hygro. Cells were selected with 500 μg/ml hygromycin starting 2 days after electroporation and transduction and with 500 ng/ml puromycin starting 8 days after electroporation and transduction. Following selection, clonal populations of the cells were isolated by limiting dilution.

### Antibodies

Mouse monoclonal anti-Tsg101 (clone C-2, catalog no. SC-7964), anti-ALIX (clone 1A12, catalog no. SC-53540), and anti-EGFR (clone A-10, catalog no. SC-373746) primary antibodies were purchased from Santa Cruz Biotechnology, Inc. (Dallas, TX). The anti-GFP primary antibody was an in-house rabbit polyclonal antibody raised against the intact GFP molecule (NICHD, National Institutes of Health). HRP-conjugated goat anti-mouse and goat anti-rabbit secondary antibodies were purchased from Invitrogen.

Mouse monoclonal anti-α-Tubulin (clone B-5-1-2, catalog no. T5168) primary antibody was purchased from Sigma-Aldrich and directly coupled to Alexa Fluor 647 NHS ester (catalog no. A20006, Life Technologies/Invitrogen) or Atto 532 NHS ester (catalog no. 88793, Sigma-Aldrich).

HIV immunoglobulin (HIV-Ig) was obtained from the National Institutes of Health AIDS Research and Reference Reagent Program. Mouse monoclonal anti-GAPDH antibody (catalog no. AM4300) was purchased from Invitrogen. Anti-human IgG conjugated to HRP was purchased from Sigma-Aldrich (catalog no. GENA933). Near-IR 800–labeled goat anti-mouse secondary antibody (catalog no. AC2135) and 488-labeled goat anti-human secondary antibody (catalog no. AC2208) were purchased from Azure Biosystems (Dublin, CA).

### Western blotting for Tsg101, GFP, and ALIX

Cells were pelleted and frozen at −80 °C and then lysed in cold radioimmune precipitation assay buffer (50 mm Tris, pH 7.4, 150 mm NaCl, 1% Triton X-100, 1 mm EDTA) supplemented with protease inhibitor mixture (Sigma-Aldrich (catalog no. S8830) or GoldBio (St. Louis, MO) (catalog no. GB-108-2)). Cell lysates were clarified by centrifugation, and the supernatants were normalized for total protein concentration according to the BCA assay (Thermo Scientific (Waltham, MA), catalog no. 23227). Proteins were resolved by SDS-PAGE on 4–20% gradient gels (Bio-Rad, catalog no. 4568096) and then transferred to nitrocellulose membranes. For the Tsg101 and ALIX Western blotting, the membranes were blocked with 5% milk in TBS, pH 7.4, and then probed with the mouse anti-Tsg101 or anti-ALIX primary antibodies (listed above) at 1:200 dilution in TBS, pH 7.4, with 5% milk and 0.05% Tween 20, followed by the goat anti-mouse HRP secondary antibody at 1:8000 dilution, and the secondary HRP conjugates were detected using ProSignal Femto ECL Reagent (Genesee Scientific (San Diego, CA), catalog no. 20-302). For the GFP Western blotting, the membranes were blocked with 5% milk in PBS, pH 7.4, and then probed with the rabbit anti-GFP primary antibody at 1:5000 dilution in PBS, pH 7.4, with 5% milk and 0.05% Tween 20, followed by the goat anti-rabbit HRP secondary antibody at 1:10,000 dilution, and the secondary HRP conjugates were detected using ProSignal Dura ECL Reagent (Genesee Scientific, catalog no. 20-301). The membranes were then reblocked and probed with anti-α-tubulin AF647 for a loading control. Western blotting images were acquired using a FluorChem imager (ProteinSimple, San Jose, CA).

### Sequencing of Tsg101 alleles

Genomic DNA (gDNA) was isolated from cells as described previously (76). The gDNA was used as template for PCR amplification of the regions of interest, and the PCR products were gel-purified and sequenced by Sanger sequencing. For the non-KI alleles, PCR was performed with forward and reverse primers that anneal in the gDNA flanking the Tsg101 sgRNA target site. This primer pair could also anneal to KI alleles, but the amplicon from those alleles would be larger by the size of the Puro-P2A-EGFP insert, so it was excluded by limiting the extension time and by gel-purifying only the amplicon band around the expected size for a non-KI allele. For the 5′ KI junctions, PCR was performed with a forward primer that anneals in the gDNA upstream of the target site and a reverse primer that anneals in the PuroR coding sequence. For the 3′ KI junctions, PCR was performed with a forward primer that anneals in the GFP coding sequence and a reverse primer that anneals in the gDNA downstream of the target site.

### Confocal microscopy

Imaging was performed with a customized inverted Nikon Ti-E microscope (Solamere Technology Group Inc., Salt Lake City, UT) using ×60, CFI Plan Apo Lambda 1.4 NA oil-immersion objectives (Nikon Instruments, Melville, NY). Fiber-coupled 488-, 514-, 561-, and 640-nm lasers (OBIS CW solid-state lasers, Coherent, Santa Clara, CA) were used in combination with a CSU-X A1 spinning disk unit (Yokogawa Electronics, Tokyo, Japan) to excite and collect confocal sections of GFP, Atto532, mCherry, and AF647 or DRAQ7 fluorescence, respectively.

Microscopy experiments used 25-mm #1.5 coverslips (Warner Instruments, Hamden, CT), which were sterilized by soaking in ethanol and air-dried before seeding cells onto them as described below.

### Midbody localization of GFP-Tsg101

For HeLa, coverslips were pretreated with fibronectin (Sigma-Aldrich, catalog no. FC010) at 1:50 dilution in DPBS for 30 min at room temperature before adding cells, which were then cultured overnight to allow cell division to occur. For Jurkat, coverslips were pretreated with poly-l-lysine (Ted Pella Inc. (Redding, CA), catalog no. 18026) for at least 5 min at room temperature and then washed with sterile H_2_O and dried overnight before adding cells, which had been passaged the previous day and were transferred without titrating the cell suspension in order to avoid disrupting cytokinetic bridges.

Cells were fixed with 4% paraformaldehyde (Electron Microscopy Sciences (Hatfield, PA), catalog no. 15710) and 0.2% glutaraldehyde (Electron Microscopy Sciences, catalog no. 16220) in DPBS for 30 min, quenched with three 5-min washes of 30 mm glycine (Sigma-Aldrich, catalog no. G8898) in DPBS, permeabilized with 0.2% Triton X-100 (Sigma-Aldrich, catalog no. T8787) in DPBS for 10 min, blocked with 10% BSA (Sigma-Aldrich, catalog no. A3983) in DPBS for 30 min, stained with anti-α-tubulin Atto532 (for the HeLa experiment) or anti-α-tubulin AF647 (for the Jurkat experiment) in DPBS for 20 min, and washed three times for 10 min each with 0.2% BSA and 0.5% Triton X-100 in DPBS.

Imaging was performed on spinning disk confocal microscopes as described above. For the HeLa experiment, Atto532 was imaged using the 514-nm laser at 15 milliwatts with 100-ms exposure, and GFP was imaged using the 488-nm laser at 10 milliwatts with 200-ms exposure and at an overall microscope magnification of ×90. For the Jurkat experiment, AF647 was imaged using the 640-nm laser at 5 milliwatts with 100-ms exposure, and GFP was imaged using the 488-nm laser at 35 milliwatts with 200-ms exposure. In both experiments, *z*-stacks at 0.2-μm spacing were collected for fields of view showing midbodies, and maximum intensity projections of the *z*-stacks were generated using ImageJ software (National Institutes of Health, Bethesda, MD). The raw images were subsequently filtered with a Gaussian smoothing filter (5 × 5-pixel volume, σ = 1 pixel), sharpened with Matlab's (Mathworks, Natick, MA) inbuilt *unsharp* function (α = 0.9), and γ-corrected (γ = 1.1).

### Multinuclearity assay

For HeLa experiment, cells were seeded onto coverslips pretreated with fibronectin as described above and cultured on the coverslips for 2 days to around 80% confluence. For Jurkat, 10^6^ cells were seeded onto coverslips on the day of the assay in medium containing 2% methylcellulose to induce adhesion. Cells were washed with DPBS and fixed, quenched, and permeabilized as described above and then stained with DRAQ7 nuclear dye (Abcam (Cambridge, UK), catalog no. ab109202) at 1:1000 dilution in DPBS for at least 5 min. Imaging was performed on a confocal microscope as described above, using the 640-nm laser. Images were acquired randomly across the coverslip and manually scored to quantify the number of multinucleate cells and total number of cells. At least 250 cells were scored for each line (*n* = 256 for KI HeLa A, *n* = 256 for KI HeLa B, *n* = 259 for WT HeLa, *n* = 258 for KI Jurkat A, *n* = 255 for KI Jurkat B, and *n* = 256 for WT Jurkat). GraphPad Prism version 5 for Windows (GraphPad Software, Inc., La Jolla, CA) was used to calculate S.E. and to assess statistical significance by one-way analysis of variance.

### MVB localization of GFP-Tsg101

For the HeLa experiment, cells were seeded onto coverslips pretreated with fibronectin as described above and then transfected the following day with a mix of mCherry-Vps4A(E228Q), Vps4A(E228Q), and Vps4B(E235Q) plasmids, using PEI transfection reagent. For the Jurkat experiment, cells were electroporated with a mix of mCherry-Vps4A(E228Q), Vps4A(E228Q), and Vps4B(E235Q) plasmids, using the Neon transfection system with a 100-μl tip, 1350-V pulse voltage, 10-ms pulse width, and 3 pulses. In both cases, imaging was performed 18–19 h after transfection on a confocal microscope as described above. For HeLa, mCherry was imaged using the 561-nm laser at 16 milliwatts, and GFP was imaged using the 488-nm laser at 17 milliwatts; for Jurkat, mCherry was imaged using the 561-nm laser at 30 milliwatts, and GFP was imaged using the 488-nm laser at 21 milliwatts. Both experiments used exposure of 200 ms. *Z*-stacks at 0.2-μm spacing were collected for fields of view showing transfected cells, and maximum intensity projections of the *z*-stacks were generated using ImageJ software (National Institutes of Health). The raw images were subsequently filtered with a Gaussian smoothing filter (5 × 5-pixel volume, σ = 1 pixel), sharpened with Matlab's (Mathworks) inbuilt *unsharp* function (α = 0.9), and γ-corrected (γ = 1.1).

### EGFR degradation assay

Two subconfluent 6-cm dishes of each HeLa cell line were serum-starved overnight, and then one of the dishes was stimulated with 100 ng/ml EGF (GoldBio, catalog no. 1150-04-100) for 180 min while the other dish was kept without EGF. The dishes were then placed on ice, and the cells were lysed in cold radioimmune precipitation assay buffer (50 mm Tris, pH 7.4, 150 mm NaCl, 1% Triton X-100, 1 mm EDTA) supplemented with protease inhibitor mixture (Sigma-Aldrich, catalog no. S8830). Cell lysates were clarified by centrifugation, and the supernatants were normalized for total protein concentration by the BCA assay (Thermo Scientific, catalog no. 23227). Proteins were resolved by SDS-PAGE on 4–20% gradient gels (Bio-Rad, catalog no. 4568096) and then transferred to nitrocellulose membranes, blocked in TBS, pH 7.5, with 5% milk and 0.05% Tween 20, and probed with anti-EGFR primary antibody (listed above) at 1:200 dilution in TBS, pH 7.5, with 5% milk and 0.05% Tween 20, followed by goat anti-mouse HRP secondary antibody at 1:6000 dilution, and the secondary HRP conjugate was detected using ProSignal Dura ECL reagent (Genesee Scientific, catalog no. 20-301). The membranes were then reblocked and probed with anti-α-tubulin AF647 for a loading control. Western blotting images were acquired using a ProteinSimple FluorChem imager. Image Studio Lite version 5.2 software (LI-COR Biosciences, Lincoln, NE) was used for the Western blotting quantification.

### Imaging recruitment to HIV-1 assembly sites

GFP-Tsg101 KI HeLa cells (clone A) were seeded onto coverslips pretreated with fibronectin as described above and then infected the following day with lentivirus of pSV-dE-CANTD-TagRFP-3′LTR. Around 40 h after infection, assembly sites were imaged live at 37 °C and with supplemented 5% CO_2_ using a custom-built TIRF microscope capable of simultaneous dual-color imaging. Fluorescence excitation of GFP and TagRFP was achieved with 473- and 561-nm diode lasers, respectively (Opto Engine, Midvale, UT). Through-objective TIRF and fluorescent light collection were achieved with a 60 × 1.49 NA apo-TIRF objective (Nikon, Melville, NY) and detected using a scientific CMOS camera (Hamamatsu Orca Flash4.0 V2+; C11440-22CU+ (Hamamatsu City, Japan)). Simultaneously acquired images of GFP-Tsg101 and Gag were cropped by hand into ROIs of single diffraction-limited co-localization events and passed to a custom Matlab (Mathworks) analysis function to analyze intensities in time. Maximum intensities per frame were found for individual stacks before being time-normalized and pooled. Frames with instances of Gag intensity at near background levels immediately before a global positive intensity derivative were normalized to *t* = 0, and a moving average filter (size = 3 data points) was applied to the mean and first confidence interval curves for clarity. Raw images were filtered with a Gaussian smoothing filter (5 × 5-pixel volume, σ = 1 pixel) and then sharpened with Matlab's (Mathworks) inbuilt *unsharp* function (α = 0.9). The subsequent images were γ-corrected (γ = 1.1).

### HIV-1 release, infectivity, and replication assays

HeLa cell transfections were performed with the Lipofectamine 2000 reagent (Thermo Fisher Scientific) according to the manufacturer's instructions. Jurkat cells were transfected with DEAE-dextran as reported previously (77). Transfected-cell supernatants were passed through a 0.45-μm pore size filter, normalized for reverse transcriptase (RT) activity (78), and used for infectivity assays in TZM-bl cells as reported previously (79).

The quantitative virus release assay has been described in detail previously (63). Briefly, cells were transfected with 2 μg of proviral plasmid DNA. After ∼24 h, supernatants were passed through a 0.45-μm pore size filter, and virions were pelleted by ultracentrifugation over a 20% (w/v) sucrose cushion for 1.25 h at 41,500 × *g* at 4 °C on a Sorvall S55-A2 fixed-angle rotor (Thermo Fisher Scientific). Cell and virus fractions were lysed in lysis buffer (30 mm NaCl, 50 mm Tris-HCl, pH 7.5, 0.5% Triton X-100, 10 mm iodoacetamide, complete protease inhibitor (Roche Applied Science)). Lysates were subjected to SDS-PAGE on 4–15% gradient gels (Bio-Rad, catalog no. 5671084), using standard Western blotting techniques as described above. HIV proteins were detected with 10 μg/ml polyclonal HIV immunoglobulin (HIV-Ig) obtained from the National Institutes of Health AIDS Research and Reference Reagent Program. Anti-human IgG conjugated to HRP was obtained from Sigma-Aldrich (catalog no. GENA933) and used at a 1:5000 dilution. A ChemiDoc imaging system (Bio-Rad) was used for chemiluminescent detection. Virus release efficiency was calculated as the amount of virion p24 divided by total Gag (cell Pr55Gag + cell p24 + virion p24).

To generate viral particles for transduction of HeLa and Jurkat, 293T cells were co-transfected with pNL4-3 molecular clone, pCMVRΔ8.2 (80), and pHCMV-G (81) at a ratio of 2:1:0.2 using Lipofectamine 2000 as per the manufacturer's instructions. 48 h post-transfection, supernatant was collected and filtered through a 0.45-μm filter and stored at −80 °C until use. This supernatant was normalized for RT activity. The day before transduction, equivalent numbers of HeLa and Jurkat cells were seeded. The following day, RT-normalized virus was added to the HeLa and Jurkat cells. 12 h after transduction, cells were washed three times with PBS and then returned to incubation. 48 h post-transduction, supernatant was collected and filtered through a 0.45-μm filter. Cell and viral lysates were subjected to SDS-PAGE on 4–15% gradient gels (Bio-Rad, catalog no. 3450027) using standard Western blotting techniques as described above. Proteins were detected with primary antibodies against GAPDH (Invitrogen, catalog no. AM4300) and HIV-Ig. A near-IR 800–labeled goat anti-mouse secondary antibody (Azure Biosystems, catalog no. AC2135) was used at a 1:10,000 dilution to detect GAPDH, and a 488-labeled goat anti-human secondary antibody (Azure Biosystems, catalog no. AC2208) was used at a 1:5000 dilution to detect HIV-Ig. Protein bands were visualized using the Sapphire Biomolecular Imager (Azure Biosystems), and virus release efficiency was calculated as described above.

### Imaging of released HIV-1 particles

To produce each of the released HIV-1 particle samples, a 10-cm dish of cells at 60–80% confluence was infected with 3.5 ml of lentivirus of pSV-dE-CANTD-TagRFP-3′LTR. 52 h after infection, the medium containing released virus was harvested from each dish, 0.45-μm-filtered, and centrifuged for 10 min at 2900 × *g* at 4 °C to remove cell debris. Virus particles were purified from the supernatant as described by Ott (82). In brief, virus particles were pelleted by ultracentrifugation on a 20% sucrose cushion for 1 h at 125,000 × *g* at 4 °C, resuspended in TNE buffer (10 mm Tris-HCl, pH 8.0, 100 mm NaCl, 2 mm EDTA), and then mixed with an equal volume of 2× digestion buffer (40 mm Tris-HCl, pH 8.0, 2 mm CaCl_2_) containing 2 mg/ml subtilisin A and incubated for 18 h at 37 °C in order for the subtilisin to digest away proteins not protected within virus particles. The subtilisin digest was quenched with 5 μg/ml PMSF for 15–20 min at room temperature, and the virus particles were isolated by a second ultracentrifugation on 20% sucrose cushion for 1 h at 125,000 × *g* at 4 °C and then resuspended in DPBS, placed on poly-l-lysine–coated Piranha-cleaned coverslips, and incubated for 3 h to adhere to the coverslips prior to imaging. For the GFP-AP2σ1 control, a HeLa cell line stably expressing GFP-AP2σ1 was generated by transducing WT HeLa cells with GFP-AP2σ1 via lentiviral vector and selecting with puromycin, and HIV-1 particles from this cell line were produced and purified by the same protocols as above.

The purified virus was imaged under TIRF illumination. The experiment to show cross-correlation of GFP-Tsg101 with HIV-1 Gag in released particles (Fig. 6, *A* and *B*) used the 561-nm laser at 170 microwatts and the 488-nm laser at 4.61 milliwatts (measured from the rear aperture of the microscope) with exposures of 50 ms, whereas the experiment to quantify HIV-1 incorporation of GFP-Tsg101 in each cell line (Fig. 6, *C* and *D*) used the 561-nm laser at 25 milliwatts and the 473-nm laser at 50 milliwatts with exposures of 200 ms. Through-objective TIRF and fluorescent light collection were achieved with a ×60 1.49 NA apo-TIRF objective (Nikon, Melville, NY) and detected using a scientific CMOS camera (Hamamatsu Orca Flash4.0 V2+; C11440–22CU+ (Hamamatsu City, Japan)). For the examples in Fig. 6*A*, the raw images were subsequently filtered with a Gaussian smoothing filter (5 × 5-pixel volume, σ = 1 pixel), sharpened with Matlab's (Mathworks) inbuilt *unsharp* function (α = 0.9), and γ-corrected (γ = 1.1).

### Cross-correlation analysis of HIV-1 particle images

Cross-correlation analysis was performed on the raw images using custom software (31) written in Matlab (Mathworks). In brief, binary images of both the TagRFP (HIV-1 Gag) and GFP (Tsg101) channels were generated using a thresholding parameter set at three S.D. values above the mean intensity of all pixels in the image, and the cross-correlation function was calculated by the following equation (83),

(Eq. 1) C=Re⁡al[FFT−1(FTT(Im⁡tRFP))×conj(FFT(Im⁡GFP))/ρtREPρGFPN(r)]

where *Real*[] represents the real part; *FFT*() *and FFT*^−1^() represent the fast Fourier transform and inverse fast Fourier transform, respectively; *conj*() represents the complex conjugate; *Im^tRFP^* and *Im^GFP^* are the binary images of the respective channels; ρ*^tRFP^* and ρ*^GFP^* are the mean number of positive pixels in the respective binary images; and *N*(*r*) is a normalization factor for the number of detected spots in each image and the size of the image. The mean radial cross-correlation function *C_r_* was calculated by radial averaging of the cross-correlation function *C* for distances *r. C_r_* values greater than 1 indicate colocalization. GraphPad Prism version 5 for Windows (GraphPad Software) was used to assess statistical significance by performing two-tailed *t* tests to compare the *C_r_* values for the virus particles from KI HeLa A against those for the virus particles from WT HeLa for the first four data points, representing distances up to 400 nm, which encompasses the size range of HIV-1 particles (84).

### Quantification of GFP intensities and number of GFP molecules per HIV-1 particle

Single HIV-1 particle images were segmented using a custom peak-finding algorithm written in Matlab (Mathworks, Natick, MA). Briefly, particle windows were detected by smoothing particle field images using a median and Gaussian filter matching the pixel-to-pixel variance and point spread function of the microscope, respectively. Peak intensity windows were selected until either three S.D. values above background or a maximum particle threshold were met. The remaining pixels in the images that were not included in single particle windows were used to estimate the average background per pixel. For photon estimation per HIV-1 particle window, the average pixel background (25 pixels total) was subtracted from the raw integrated intensity of a 5 × 5-pixel window and then converted to photons using the factory-calibrated photon transfer curve (Hamamatsu Photonics). Segmentation of images for purified GFP molecules was treated similarly using the algorithm above.

For the single GFP molecule standard, recombinantly produced GFP-His_6_ was affinity-purified with nickel-nitrilotriacetic acid (GoldBio H-350-5) and placed on coverslips coated with 0.1% poly-l-lysine that were blocked with 4% BSA in PBS for 1 h. Single GFP molecules were imaged by TIRF-M at the same settings as described above for HIV-1 particles. The distribution of GFP ROI integrated intensities was fit to an exponential curve to obtain mean and S.D. A conservative range for single GFP molecule intensity was determined to be the mean ± S.D. Using the extrapolated single molecule GFP intensity range, the number of GFP-Tsg101 molecules incorporated into HIV-1 particles was approximated. Intensity ranges for virus particle–associated GFP-Tsg101 were determined to be most accurately described by the mean (lower limit) to the mean + 3 S.D. (upper limit) by fitting to a log-normal distribution. The total number of GFP-Tsg101 molecules per HIV-1 particle was determined by dividing the range of GFP-Tsg101 intensities by the range of single GFP molecule intensities, where the lowest GFP photon count is the divisor for the largest photon count for GFP-Tsg101 and likewise. This results in a large conservative range for the number of GFP-Tsg101 molecules incorporated into HIV-1 particles for each individual KI producer line. The method was repeated for HIV-1 particles produced from HeLa cells stably expressing GFP-AP2σ1.

For the single-step GFP photobleaching representation, three single 5 × 5 enhanced GFP photobleaching movies were randomly selected to demonstrate the robustness of the single molecule standard used. The normalized integrated intensities for these ROIs were plotted in time to show single-step photobleaching events, which are a classical indication of single-molecule fluorescence. Montage frames were smoothed in FIJI (85) using the default parameters post-analysis for improved esthetics.

## Author contributions

H. K. H., M. V. F., N. S. G., E. O. F., and S. B. v. E. conceptualization; H. K. H., M. V. F., and N. S. G. data curation; H. K. H., N. S. G., and S. B. v. E. software; H. K. H., M. V. F., and N. S. G. formal analysis; H. K. H., E. O. F., and S. B. v. E. supervision; H. K. H. and S. B. v. E. funding acquisition; H. K. H., M. V. F., and N. S. G. validation; H. K. H., M. V. F., N. S. G., E. O. F., and S. B. v. E. investigation; H. K. H., M. V. F., N. S. G., and S. B. v. E. visualization; H. K. H., M. V. F., N. S. G., E. O. F., and S. B. v. E. methodology; H. K. H., M. V. F., and S. B. v. E. writing-original draft; H. K. H., M. V. F., N. S. G., E. O. F., and S. B. v. E. writing-review and editing; E. O. F. and S. B. v. E. resources.

## Supplementary Material

Supporting Information

## Footnotes

2 The abbreviations used are:

ESCRT: endosomal sorting complexes required for transport
MVB: multivesicular body
FP: fluorescent protein
DSB: double-strand break
HDR: homology-directed repair
NHEJ: nonhomologous end joining
KI: knock-in
indel: insertion or deletion
sgRNA: single guide RNA
EGFR: epidermal growth factor receptor
TIRF-M: total internal reflection fluorescence microscopy
HR: homology region
GAPDH: glyceraldehyde-3-phosphate dehydrogenase
gDNA: genomic DNA
NA: numerical aperture
DPBS: Dulbecco's PBS
ROI: region of interest
RT: reverse transcriptase.
