# Supplementary material for: Genomic tagging of endogenous human ESCRT-I complex preserves ESCRT-mediated membrane-remodeling functions
Source: J Biol Chem. 2019 Sep 13;294(44):16266–81. doi: 10.1074/jbc.RA119.009372 (PMC6827313; doi:10.1074/jbc.RA119.009372)
Supplement: Supporting Information [file supp_294_44_16266__index.html]

Genomic tagging of endogenous human ESCRT-I complex preserves ESCRT-mediated membrane-remodeling functions — ESCRT-I knock-in cell lines — Genomic tagging of endogenous human ESCRT-I complex preserves ESCRT-mediated membrane-remodeling functions — ESCRT-I knock-in cell lines — Supporting Information 

# Genomic tagging of endogenous human ESCRT-I complex preserves ESCRT-mediated membrane-remodeling functions

## Supporting Information

- Supporting Information (to be published online) - Supplementary Figures
