# Supplementary material for: Genomic tagging of endogenous human ESCRT-I complex preserves ESCRT-mediated membrane-remodeling functions
Source: J Biol Chem. 2019 Sep 13;294(44):16266–81. doi: 10.1074/jbc.RA119.009372 (PMC6827313; doi:10.1074/jbc.RA119.009372)
Supplement: Supporting Information [file supp_RA119.009372_152856_2_supp_394301_pxrqp9.pdf]

Supplementary Figures

Genomic tagging of endogenous human ESCRT-I complex preserves ESCRT-mediated membrane remodeling functions

Huxley K. Hoffman[1], Melissa V. Fernandez[2], Nicholas S. Groves[1], Eric O. Freed[2], Schuyler B. van Engelenburg[1*]

[1] Molecular and Cellular Biophysics Program, Department of Biological Sciences, University of Denver, Denver, CO 80210, USA
[2] Virus-Cell Interaction Section, HIV Dynamics and Replication Program, Center for Cancer Research, National Cancer Institute, Frederick, MD 21702, USA

(A) Tsg101 sgRNA target site

M A V S E S Q L K K M V *S K	Tsg101 exon 1
cggccgtcatggcggtgtcggagagccagctcaagaaaatggtgt*ccaaggtgaggctg	genomic DNA
GCTCAAGAAAATGGTGT*CCA*AGG*	sgRNA

(B) GFP-Tsg101 HDR template sequence

5’ homology region, PuroR, P2A, EGFP, 3’ homology region (with **silent mutations**)

Gggtgggtacagaggagaaattctgacttacggaatgatttcctggagaagaaattactatctggcttcttgtgaaacaaaggctctacacttta
cccttgccaacgtatagtatgaaggtctttaagcttaaaaaaaatcattaattagaggaccactgggcatcatctaaggcaagtgtatgacttta
gagtcgggagaaatttgcgcctgagctagaaatgcactagcctaccattatcacaagagcaagtcagtgacagaacagttctctgaccgccagtg
cagtgccctcccacttgatcaatcagtcattaaggctcactggcaagcccttcgtacttcttccctctccggcactagcttccttaagtcgactc
tgtgaaatacctgctaatcttatcctgaaccagagtttcgaccctcggaacagtcttgcccacatctttgggacccgattcgtcctgctgattcc
ggcactcgaggggcgggttggttggaacccaccccaccaggcccctctcaatcccacacggtgtccacccttgcccgcaggacggcggcccggaa
gtgacgtagtggaagcggaagtggtgtagtggtgccgacttcctgttgtttgaggccgggttgggggtgtgcgattgtgtgggacggtctggggc
agcccagcagcggctgaccctctgcctgcggggaagggagtcgccaggcggccgtcatgaccgagtacaagcccacggtgcgcctcgccacccgc
gacgacgtccccagggccgtacgcaccctcgccgccgcgttcgccgactaccccgccacgcgccacaccgtcgatccggaccgccacatcgagcg
ggtcaccgagctgcaagaactcttcctcacgcgcgtcgggctcgacatcggcaaggtgtgggtcgcggacgacggcgcggccgtggcggtctgga
ccacgccggagagcgtcgaagcgggggcggtgttcgccgagatcggcccgcgcatggccgagttgagcggttcccggctggccgcgcagcaacag
atggaaggcctcctggcgccgcaccggcccaaggagcccgcgtggttcctggccaccgtcggagtctcgcccgaccaccagggcaagggtctggg
cagcgccgtcgtgctccccggagtggaggcggccgagcgcgccggggtgcccgccttcctggagacctccgcgccccgcaacctccccttctacg
agcggctcggcttcaccgtcaccgccgacgtcgaggtgcccgaaggaccgcgcacctggtgcatgacccgcaagcccggtgccggcagcggcgcc
accaacttcagcctgctgaagcaggccggcgacgtggaggagaaccccggccccatggtgagcaagggcgaggagctgttcaccggggtggtgcc
catcctggtcgagctggacggcgacgtaaacggccacaagttcagcgtgtccggcgagggcgagggcgatgccacctacggcaagctgaccctga
agttcatctgcaccaccggcaagctgcccgtgccctggcccaccctcgtgaccaccctgacctacggcgtgcagtgcttcagccgctaccccgac
cacatgaagcagcacgacttcttcaagtccgccatgcccgaaggctacgtccaggagcgcaccatcttcttcaaggacgacggcaactacaagac
ccgcgccgaggtgaagttcgagggcgacaccctggtgaaccgcatcgagctgaagggcatcgacttcaaggaggacggcaacatcctggggcaca
agctggagtacaactacaacagccacaacgtctatatcatggccgacaagcagaagaacggcatcaaggtgaacttcaagatccgccacaacatc
gaggacggcagcgtgcagctcgccgaccactaccagcagaacacccccatcggcgacggccccgtgctgctgcccgacaaccactacctgagcac
ccagtccgccctgagcaaagaccccaacgagaagcgcgatcacatggtcctgctggagttcgtgaccgccgccgggatcactctcggcatggacg
agctgtacaagtccggaatggcggtgtcggagagccagctcaa**a**aa**g**atggt**cag**caaggtgaggctgcgacgcgctcgcctcccagggcgcgcc
caccgctcccttccgcgccctgtcgagtccgtcccggcccagccaagcaagcttcccagacgggccggaagccccggtgcagtccttagcgacct
cctcagaaccccgccccgaggcgcctgtcgcctggtgcgggaatccccgtacgggagctgggagggtgggggacggcgacagtcaacaaaggcgt
ggagcggaggctacctgacacctgccgcccacccgccctcctctcttccactgagtttggagctgtctgtggggcagtgtagtttttcgtttgtt
tttataaacacaaacaagggatcatacttagttgtagatctgaggcaatcctctactcctgtcccaatctagggatcctccagtcttagcctttc
ttttgccgacccgcagagagtgtggccttttcagttttgtattctccagaaaagaagcgaaaataccagtcccttactgccccaggtgaaataca
taccattaccatttcagggattttctaagagtaactacttttaagtgtatgcttcttataacgcgtatttatttgctttgagcttaatttatgat
cttcacggctcaag

Figure S1. Guide RNA and HDR donor template sequences for GFP-Tsg101 knock-in. (A) Tsg101 sgRNA and its target site in Tsg101 Exon 1. The protospacer sequence is underlined and the protospacer adjacent motif (PAM) is *italicized*. The Cas9 nuclease cuts the DNA 3-4 nucleotides upstream of the PAM (*). (B) Annotated sequence of the GFP-Tsg101 HDR template, containing a PuroR-P2A-EGFP cassette flanked by homology regions matching the genomic DNA on either side of the target site.

**WT reference sequence**	ATGGCGGTGTCGGAGAGCCAGCTCAAGAAAATGGTGT*CCAAGGTGAGGCTGCGACGCGCTCGCCTCCCAG<br>**M A V S E S Q L K K M V *S K**	(*) sgRNA target site.<br>**Translation: Tsg101 Exon 1.**
**KI HeLa A**	ATGGCGGTGTCGGAGAGCCAGCTCAAGA*TGAGGCTGCGACGCGCTCGCCTCCCAG<br>**M A V S E S Q L K M R L R R A R L P**<br>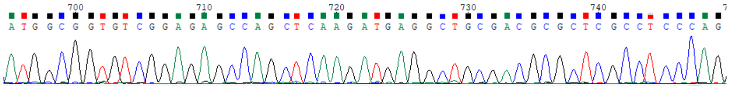	*** 15 nt deletion removes splice site.**
**KI HeLa B**	ATGGCGGTGTCGGAGAGCCAGCTCAAGAAAATGGTGTGCCAAGGTGAGGCTGCGACGCGCTCGCCTCCCAG<br>**M A V S E S Q L K K M V C Q G E A A T R S P P**<br>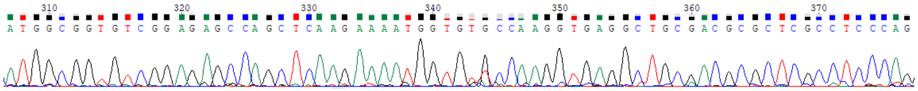	**1 nt insertion causes frameshift.**
**KI Jurkat A**	ATGGCGGTGTCGGAGAGCCAGCTCAAGAAAATGGTGTAGCCCCCAAGGTGAGGCTGCGACGCGCTCGCCTCCCAG<br>**M A V S E S Q L K K M V -**<br>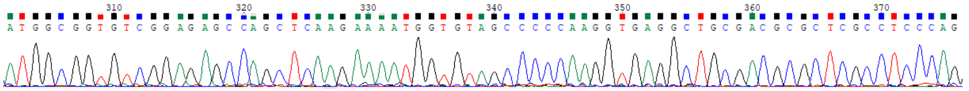	**5 nt insertion introduces premature stop codon.**
**KI Jurkat B**	ATGGCGGTGTCGGAGAGCCAGCTCAAGAAAATGGTGTAGCCCCCAAGGTGAGGCTGCGACGCGCTCGCCTCCCAG<br>**M A V S E S Q L K K M V -**<br>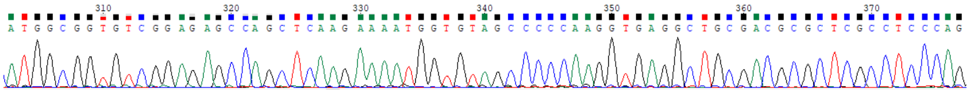	**5 nt insertion introduces premature stop codon.**

Figure S2. GFP-Tsg101 knock-in cell lines have knockout indels on their untagged Tsg101 alleles. To sequence the non-knock-in alleles of Tsg101, PCR was performed on genomic DNA of the knock-in cell lines with primers that anneal to the genomic DNA on either side of the Tsg101 target site. The primers could also anneal to a knock-in allele, but that would give a much larger PCR amplicon, which was excluded by limiting the extension time and by gel-purifying the band at the predicted size for a non-knock-in allele. Sanger sequencing of the PCR products showed indels that knock-out expression of Tsg101. The indels, and their effects on the translated protein sequence, are indicated in red. This confirms that the knock-in cell lines have no functional alleles for untagged Tsg101.

(A) 5' knock-in junction

```
HumanGenomeRef     1 GATTCGTCCTGCTGATTCCGGCACTCGAGGGGCGGGTTGGTTGGAACCCACCCCACCAGGCCCCTCTCAATCCCACACGG
DonorPlasmid       1 GATTCGTCCTGCTGATTCCGGCACTCGAGGGGCGGGTTGGTTGGAACCCACCCCACCAGGCCCCTCTCAATCCCACACGG
WT_HeLa            1 GATTCGTCCTGCTGATTCCGGCACTCGAGGGGCGGGTTGGTTGGAACCCACCCCGCCAGGCCCCTCTCAATCCCACACGG
KI_HeLa_A          1 GATTCGTCCTGCTGATTCCGGCACTCGAGGGGCGGGTTGGTTGGAACCCACCCCGCCAGGCCCCTCTCAATCCCACACGG
KI_HeLa_B          1 GATTCGTCCTGCTGATTCCGGCACTCGAGGGGCGGGTTGGTTGGAACCCACCCCGCCAGGCCCCTCTCAATCCCACACGG
WT_Jurkat          1 GATTCGTCCTGCTGATTCCGGCACTCGAGGGGCGGGTTGGTTGGAACCCACCCCGCCAGGCCCCTCTCAATCCCACACGG
KI_Jurkat_A        1 GATTCGTCCTGCTGATTCCGGCACTCGAGGGGCGGGTTGGTTGGAACCCACCCCGCCAGGCCCCTCTCAATCCCACACGG
KI_Jurkat_B        1 GATTCGTCCTGCTGATTCCGGCACTCGAGGGGCGGGTTGGTTGGAACCCACCCCGCCAGGCCCCTCTCAATCCCACACGG

HumanGenomeRef    81 TGTCCACCCTTGCCCGCAGGACGGCGGCCCGGAAGTGACGTAGTGGAAGCGGAAGTGGTGTAGTGGTGCCGACTTCCTGT
DonorPlasmid      81 TGTCCACCCTTGCCCGCAGGACGGCGGCCCGGAAGTGACGTAGTGGAAGCGGAAGTGGTGTAGTGGTGCCGACTTCCTGT
WT_HeLa           81 TGTCCACCCTTGCCCGCAGGACGGCGGCCCGGAAGTGACGTAGTGGAAGCGGAAGTGGTGTAGTGGTGCCGACTTCCTGT
KI_HeLa_A         81 TGTCCACCCTTGCCCGCAGGACGGCGGCCCGGAAGTGACGTAGTGGAAGCGGAAGTGGTGTAGTGGTGCCGACTTCCTGT
KI_HeLa_B         81 TGTCCACCCTTGCCCGCAGGACGGCGGCCCGGAAGTGACGTAGTGGAAGCGGAAGTGGTGTAGTGGTGCCGACTTCCTGT
WT_Jurkat         81 TGTCCACCCTTGCCCGCAGGACGGCGGCCCGGAAGTGACGTAGTGGAAGCGGAAGTGGTGTAGTGGTGCCGACTTCCTGT
KI_Jurkat_A       81 TGTCCACCCTTGCCCGCAGGACGGCGGCCCGGAAGTGACGTAGTGGAAGCGGAAGTGGTGTAGTGGTGCCGACTTCCTGT
KI_Jurkat_B       81 TGTCCACCCTTGCCCGCAGGACGGCGGCCCGGAAGTGACGTAGTGGAAGCGGAAGTGGTGTAGTGGTGCCGACTTCCTGT

HumanGenomeRef   161 TGTTTGAGGCCGGGTTGGGGGTGTGCGATTGTGTGGGACGGTCTGGGGCAGCCCAGCAGCGGCTGACCCTCTGCCTGCGG
DonorPlasmid     161 TGTTTGAGGCCGGGTTGGGGGTGTGCGATTGTGTGGGACGGTCTGGGGCAGCCCAGCAGCGGCTGACCCTCTGCCTGCGG
WT_HeLa          161 TGTTTGAGGCCGGGTTGGGGGTGTGCGATTGTGTGGGACGGTCTGGGGCAGCCCAGCAGCGGCTGACCCTCTGCCTGCGG
KI_HeLa_A        161 TGTTTGAGGCCGGGTTGGGGGTGTGCGATTGTGTGGGACGGTCTGGGGCAGCCCAGCAGCGGCTGACCCTCTGCCTGCGG
KI_HeLa_B        161 TGTTTGAGGCCGGGTTGGGGGTGTGCGATTGTGTGGGACGGTCTGGGGCAGCCCAGCAGCGGCTGCCCCTCTGCCTGCGG
WT_Jurkat        161 TGTTTGAGGCCGGGTTGGGGGTGTGCGATTGTGTGGGACGGTCTGGGGCAGCCCAGCAGCGGCTGACCCTCTGCCTGCGG
KI_Jurkat_A      161 TGTTTGAGGCCGGGTTGGGGGTGTGCGATTGTGTGGGACGGTCTGGGGCAGCCCAGCAGCGGCTGACCCTCTGCCTGCGG
KI_Jurkat_B      161 TGTTTGAGGCCGGGTTGGGGGTGTGCGATTGTGTGGGACGGTCTGGGGCAGCCCAGCAGCGGCTGACCCTCTGCCTGCGG

HumanGenomeRef   241 GGAAGGGAGTCGCCAGGCGGCCGTCATG---------------
DonorPlasmid     241 GGAAGGGAGTCGCCAGGCGGCCGTCATGACCGAGTACAAGCCC
WT_HeLa          241 GGAAGGGAGTCGCCAGGCGGCCGTCATG---------------
KI_HeLa_A        241 GGAAGGGAGTCGCCAGGCGGCCGTCATGACCGAGTACAAGCCC
KI_HeLa_B        241 GGAAGGGAGTCGCCAGGCGGCCGTCATGACCGAGTACAAGCCC
WT_Jurkat        241 GGAAGGGAGTCGCCAGGCGGCCGTCATG---------------
KI_Jurkat_A      241 GGAAGGGAGTCGCCAGGCGGCCGTCATGACCGAGTACAAGCCC
KI_Jurkat_B      241 GGAAGGGAGTCGCCAGGCGGCCGTCATGACCGAGTACAAGCCC
```

(B) 3' knock-in junction

```
HumanGenomeRef     1 ---------------------------------------ATGGCGGTGTCGGAGAGCCAGCTCAAGAAAATGGTGTCCAA
DonorPlasmid       1 GGGATCACTCTCGGCATGGACGAGCTGTACAAGTCCGGAATGGCGGTGTCGGAGAGCCAGCTCAAAAAGATGGTCAGCAA
WT_HeLa            1 ---------------------------------------ATGGCGGTGTCGGAGAGCCAGCTCAAGAAAATGGTGTCCAA
KI_HeLa_A          1 GGGATCACTCTCGGCATGGACGAGCTGTACAAGTCCGGAATGGCGGTGTCGGAGAGCCAGCTCAAAAAGATGGTCAGCAA
KI_HeLa_B          1 GGGATCACTCTCGGCATGGACGAGCTGTACAAGTCCGGAATGGCGGTGTCGGAGAGCCAGCTCAAAAAGATGGTCAGCAA
WT_Jurkat          1 ---------------------------------------ATGGCGGTGTCGGAGAGCCAGCTCAAGAAAATGGTGTCCAA
KI_Jurkat_A        1 GGGATCACTCTCGGCATGGACGAGCTGTACAAGTCCGGAATGGCGGTGTCGGAGAGCCAGCTCAAAAAGATGGTCAGCAA
KI_Jurkat_B        1 GGGATCACTCTCGGCATGGACGAGCTGTACAAGTCCGGAATGGCGGTGTCGGAGAGCCAGCTCAAAAAGATGGTCAGCAA

HumanGenomeRef    42 GGTGAGGCTGCGACGCGCTCGCCTCCCAGGGCGCGCCCACCGCTCCCTTCCGCGCCCTGTCGAGTCCGTCCCGGCCCAGC
DonorPlasmid      81 GGTGAGGCTGCGACGCGCTCGCCTCCCAGGGCGCGCCCACCGCTCCCTTCCGCGCCCTGTCGAGTCCGTCCCGGCCCAGC
WT_HeLa           42 GGTGAGGCTGCGACGCGCTCGCCTCCCAGGGCGCGCCCACCGCTCCCTTCCGCGCCCTGTCGAGTCCGTCCCGGCCCAGC
KI_HeLa_A         81 GGTGAGGCTGCGACGCGCTCGCCTCCCAGGGCGCGCCCACCGCTCCCTTCCGCGCCCTGTCGAGTCCGTCCCGGCCCAGC
KI_HeLa_B         81 GGTGAGGCTGCGACGCGCTCGCCTCCCAGGGCGCGCCCACCGCTCCCTTCCGCGCCCTGTCGAGTCCGTCCCGGCCCAGC
WT_Jurkat         42 GGTGAGGCTGCGACGCGCTCGCCTCCCAGGGCGCGCCCACCGCTCCCTTCCGCGCCCTGTCGAGTCCGTCCCGGCCCAGC
KI_Jurkat_A       81 GGTGAGGCTGCGACGCGCTCGCCTCCCAGGGCGCGCCCACCGCTCCCTTCCGCGCCCTGTCGAGTCCGTCCCGGCCCAGC
KI_Jurkat_B       81 GGTGAGGCTGCGACGCGCTCGCCTCCCAGGGCGCGCCCACCGCTCCCTTCCGCGCCCTGTCGAGTCCGTCCCGGCCCAGC

HumanGenomeRef   122 CAAGCAAGCTTCCCAGACGGGCCGGAAGCCCCGGTGCAGTCCTTAGCGACCTCCTCAGAACCCCGCCCCGAGGCGCCTGT
DonorPlasmid     161 CAAGCAAGCTTCCCAGACGGGCCGGAAGCCCCGGTGCAGTCCTTAGCGACCTCCTCAGAACCCCGCCCCGAGGCGCCTGT
WT_HeLa          122 CAAGCAAGCTTCCCAGACGGGCCGGAAGCCCCGGTGCAGTCCTTAGCGACCTCCTCAGAACCCCGCCCCGAGGCGCCTGT
KI_HeLa_A        161 CAAGCAAGCTTCCCAGACGGGCCGGAAGCCCCGGTGCAGTCCTTAGCCACCTCCTCAGACCCCCGCCCCGAGGCGCCTGT
KI_HeLa_B        161 CAAGCAAGCTTCCCAGACGGGCCGGAAGCCCCGGTGCAGTCCTTAGCCACCTCCTCAGACCCCCGCCCCGAGGCGCCTGT
WT_Jurkat        122 CAAGCAAGCTTCCCAGACGGGCCGGAAGCCCCGGTGCAGTCCTTAGCCACCTCCTCAGAACCCCGCCCCGAGGCGCCTGT
KI_Jurkat_A      161 CAAGCAAGCTTCCCAGACGGGCCGGAAGCCCCGGTGCAGTCCTTAGCGACCTCCTCAGAACCCCGCCCCGAGGCGCCTGT
KI_Jurkat_B      161 CAAGCAAGCTTCCCAGACGGGCCGGAAGCCCCGGTGCAGTCCTTAGCGACCTCCTCAGAACCCCGCCCCGAGGCGCCTGT

HumanGenomeRef   202 CGCCTGGTGCGGGAATCCCCGTACGGGAGCTGGGAGGGTGGGGGACGGCGACAGTCAACAAAGGCGTGGAGCGGAGGCTA
DonorPlasmid     241 CGCCTGGTGCGGGAATCCCCGTACGGGAGCTGGGAGGGTGGGGGACGGCGACAGTCAACAAAGGCGTGGAGCGGAGGCTA
WT_HeLa          202 CGCCTGGTGCGGGAATCCCCGTACGGGAGCTGGGAGGGTGGGGGACGGCGACAGTCAACAAAGGCGTGGAGCGGAGGCTA
KI_HeLa_A        241 CGCCTGGTGCGGGAATCCCCGTACGGGAGCTGGGAGGGTGGGGGACGGCGACAGTCAACAAAGGCGTGGAGCGGAGGCTA
KI_HeLa_B        241 CGCCTGGTGCGGGAATCCCCGTACGGGAGCTGGGAGGGTGGGGGACGGCGACAGTCAACAAAGGCGTGGAGCGGAGGCTA
WT_Jurkat        202 CGCCTGGTGCGGGAATCCCCGTACGGGAGCTGGGAGGGTGGGGGACGGCGACAGTCAACAAAGGCGTGGAGCGGAGGCTA
KI_Jurkat_A      241 CGCCTGGTGCGGGAATCCCCGTACGGGAGCTGGGAGGGTGGGGGACGGCGACAGTCAACAAAGGCGTGGAGCGGAGGCTA
KI_Jurkat_B      241 CGCCTGGTGCGGGAATCCCCGTACGGGAGCTGGGAGGGTGGGGGACGGCGACAGTCAACAAAGGCGTGGAGCGGAGGCTA

HumanGenomeRef   282 CCTGACACCTGCCGCCCACCCGCCCTCCTCTCTTCCACTGAGTTTGGAGCTGTCTGTGGGGC
DonorPlasmid     321 CCTGACACCTGCCGCCCACCCGCCCTCCTCTCTTCCACTGAGTTTGGAGCTGTCTGTGGGGC
WT_HeLa          282 CCTGACACCTGCCGCCCACCCGCCCTCCTCTCTTCCACTGAGTTTGGAGCTGTCTGTGGGGC
KI_HeLa_A        321 CCTGACACCTGCCGCCCACCCGCCCTCCTCTCTTCCACTGAGTTTGGAGCTGTCTGTGGGGC
KI_HeLa_B        321 CCTGACACCTGCCGCCCACCCGCCCTCCTCTCTTCCACTGAGTTTGGAGCTGTCTGTGGGGC
WT_Jurkat        282 CCTGACACCTGCCGCCCACCCGCCCTCCTCTCTTCCACTGAGTTTGGAGCTGTCTGTGGGGC
KI_Jurkat_A      321 CCTGACACCTGCCGCCCACCCGCCCTCCTCTCTTCCACTGAGTTTGGAGCTGTCTGTGGGGC
KI_Jurkat_B      321 CCTGACACCTGCCGCCCACCCGCCCTCCTCTCTTCCACTGAGTTTGGAGCTGTCTGTGGGGC
```

Figure S3. GFP-Tsg101 knock-in alleles show precise insertion. (A) 5' and (B) 3' homology junctions of the KI alleles were amplified by PCR from genomic DNA, analyzed by Sanger sequencing, and compared to the human genome reference sequence, donor plasmid sequence, and sequences from the parental (WT) cell lines. Non-coding regions (5'UTR and intron) are shown in black/white text, Tsg101 exon 1 in blue, PuroR in red, and GFP in green. The results show precise integration of the PuroR-P2A-GFP insert, with no abnormalities except for several single-nucleotide polymorphisms in non-coding regions, some of which were also found in the parental lines. The discrepancies in the Tsg101 coding sequence in the alignment are the silent mutations intentionally introduced to the donor plasmid to prevent CRISPR from cutting the knock-in allele.

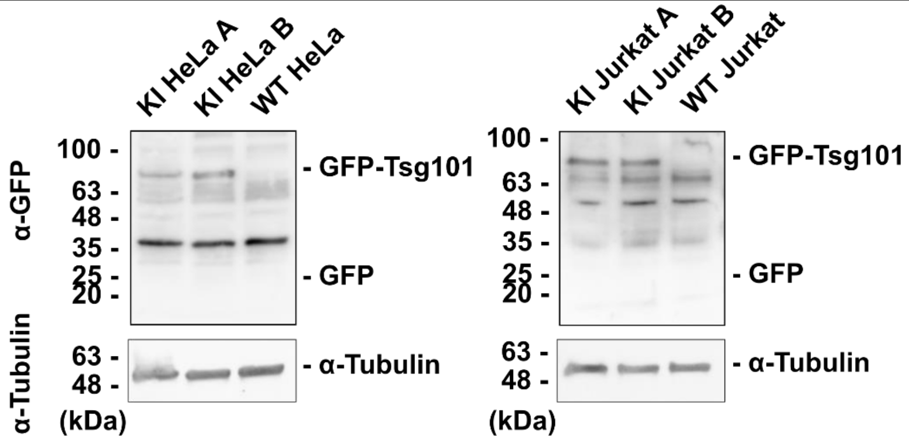

Figure S4. GFP-Tsg101 knock-in cell lines express GFP-Tsg101 with no free GFP. Western blotting for GFP in cell lysates showed that all GFP in the KI cell lines was the GFP-Tsg101 fusion protein, with no free GFP detected. The additional bands, which were detected in the wild-type (WT) parental lines as well as in the GFP-Tsg101 KI lines, represent nonspecific binding of the antibody.

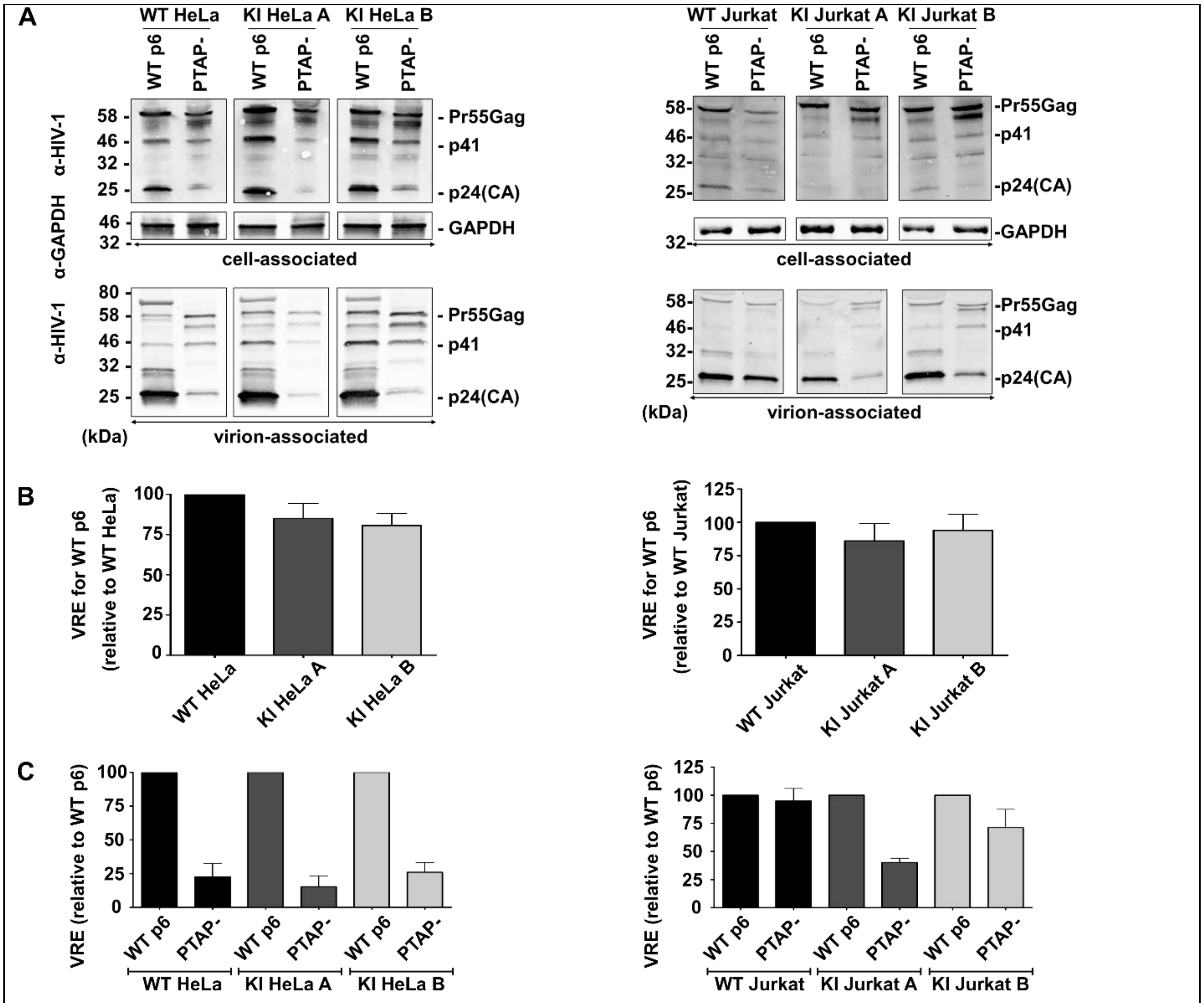

Figure S5. Viral release assays performed via transduction confirm that HIV-1 release and its dependence on Tsg101 are not disrupted in GFP-Tsg101 KI cell lines. As an alternative approach for assessing HIV-1 particle release, HeLa and Jurkat cells were transduced as described in the methods. (A) Virus release assays, as described in Fig. 5. (B) Virus release efficiencies (VRE) for WT p6 virus, calculated as in Fig. 5, normalized to the results for the WT parental cell lines; n = 3, ± s.d. (C) VRE for WT p6 or PTAP- virus, normalized to the results for WT p6; n = 3, ± s.d.

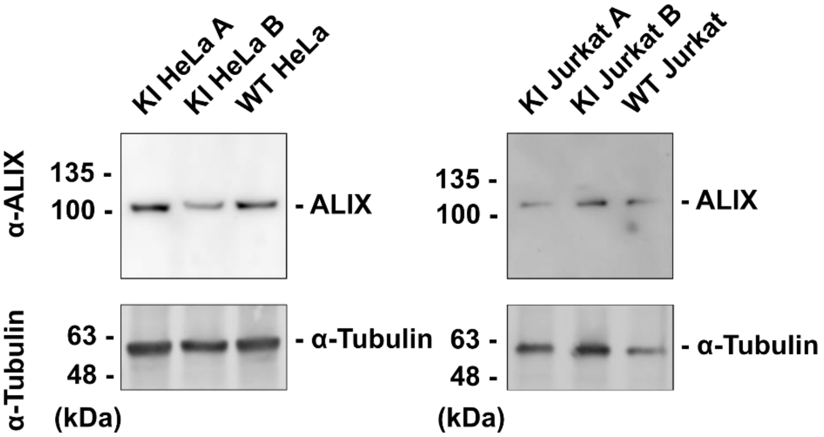

Figure S6. GFP-Tsg101 knock-in cell lines have not upregulated ALIX. The ESCRT-associated protein ALIX serves as an alternate arm of recruitment for ESCRT-III, bypassing ESCRT-I, such that ALIX overexpression can rescue some of the defects caused by loss of Tsg101 function. Western blotting for ALIX in cell lysates showed that the GFP-Tsg101 KI cell lines do not overexpress ALIX as compared to the WT parental lines, helping to rule out the possibility that the KI cells could be relying on ALIX in place of Tsg101.

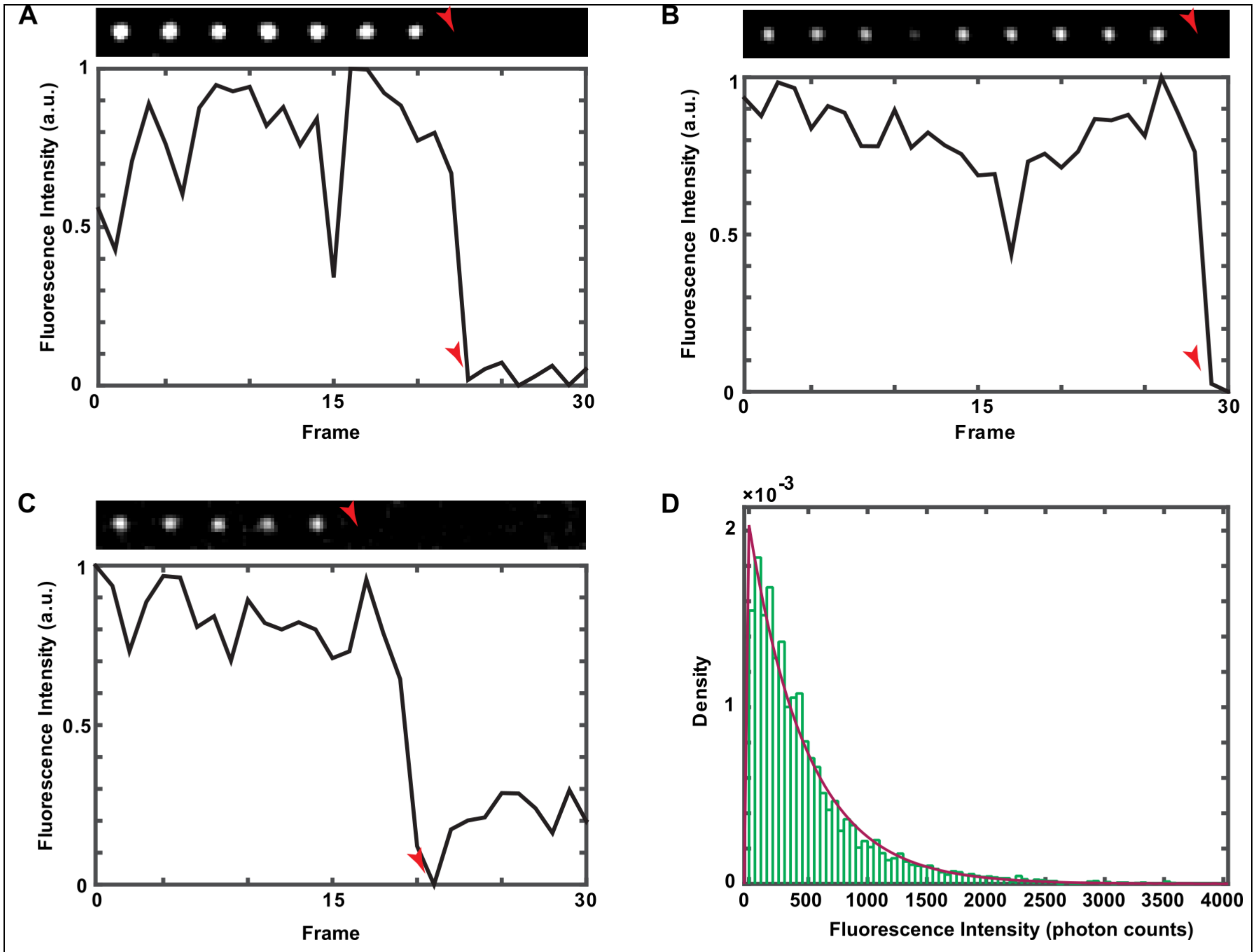

Figure S7. Single-step photobleaching of single GFP molecules to determine GFP photon counts per molecule. Recombinantly produced and affinity-purified GFP protein was placed on coverslips and imaged by TIRF-M under the same conditions as for the HIV-1 particles in Fig. 6C. Photobleaching events were acquired at 5 fps. (A-C) Examples of single-step photobleaching of GFP, with normalized fluorescence intensities plotted in time; red arrows indicate photobleaching events. Single-step photobleaching demonstrates that the GFP particles imaged represent single molecules of GFP. (D) The distribution of single molecule GFP photon counts was fit to an exponential curve to calculate upper and lower bounds (± 1 s.d.) around the mean: single GFP molecule = 231-389 photons. These upper and lower bounds for the numbers of photon counts per GFP molecule were used to calculate the lower and upper bounds respectively for the numbers of GFP molecules per HIV-1 particle in Fig. 6D.
